# Biotic factors dominate carbon fluxes and budget of a drip-irrigated and plastic-mulched cotton system in an arid oasis of Northwest China

**DOI:** 10.3389/fpls.2026.1884064

**Published:** 2026-08-13

**Authors:** Jin Wang, ChunXia Wu, Jie Bai, MingFeng Yang, Jinfeng He, YongBo Zhao, JunHai Wang, Liangbing Rong, Gang Yin

**Affiliations:** 1 Wulanwusu Agro-meteorological Experiment Station, Shihezi, China; 2 Shihezi Meteorological Bureau, Shihezi, China; 3 State Key Laboratory of Ecological Safety and Sustainable Development in Arid Lands, Xinjiang Institute of Ecology and Geography, Chinese Academy of Sciences, Urumqi, China; 4 Xinjiang Key Laboratory of Remote Sensing and Geographic Information System Applications, Urumqi, China; 5 College of Resources and Environment, University of Chinese Academy of Sciences, Beijing, China; 6 Urumqi Meteorological Satellite Ground Station, Urumqi, China; 7 Institute of Agricultural Resources and Environment, Xinjiang Academy of Agricultural Sciences, Urumqi, China; 8 College of Geography and Remote Sensing Sciences, Xinjiang University, Urumqi, China

**Keywords:** arid region, biotic factors, cotton, drip-irrigated and plastic-mulched, eddy covariance, net ecosystem carbon budget, straw return

## Abstract

Drip-irrigated and plastic-mulched (DIPM) cotton systems are widely adopted in the arid oases of Xinjiang, where water scarcity poses significant challenges to crop production. Despite the widespread use of these systems, the impact of agricultural management practices, crop development, and environmental factors on carbon fluxes and budget in these arid regions remains insufficiently quantified. This study combined eddy-covariance measurements with micrometeorology, crop growth and agricultural management to assess seasonal and interannual carbon fluxes and carbon budget over ten growing seasons (2009-2013, 2016–2019 and 2022). Net ecosystem exchange (NEE), representing the balance between carbon release (R_eco_) and carbon uptake (GPP), showed a clear source-to-sink transition, with net uptake peaking during the reproductive period, especially at the flowering stage. Seasonal NEE for the DIPM cotton system ranged from -334.07 to -431.62 g C m^-2^, with the flowering stage contributing about 61.66% of seasonal GPP and 77.77% of seasonal NEE. Biotic factors, particularly canopy development and biomass accumulation, dominated carbon fluxes during vegetative and early reproductive stages, while temperature, vapor pressure deficit and soil moisture played more influential roles during late reproduction. Carbon budget accounting revealed a trade-off: DIPM increased harvest carbon export but reduced residue return. Despite this, straw return remained the key management practice sustaining a strongly positive net ecosystem carbon budget (NECB) of 332.64 ± 57.52 g C m^-2^, with straw carbon return of 189.64 ± 46.69 g C m^-2^ and cotton-seed carbon removal of 214.89 ± 28.59 g C m^-2^. Overall, biotic factors, particularly crop development during flowering, dominate carbon gains in the DIPM cotton system, with straw return serving as the essential management lever for sustaining the net carbon sink.

## 1 Introduction

Terrestrial ecosystems play a crucial role in regulating the global carbon cycle, absorbing approximately 30% of anthropogenic CO_2_ emissions (Friedlingstein et al., 2025). Croplands, covering about 13% of the global land surface, are among the most intensively managed ecosystems and contribute significantly to uncertainties in regional and global carbon budgets (Snyder et al., 2009; Smith et al., 2010). Agricultural practices such as irrigation, mulching, fertilization, tillage, and residue management can substantially alter carbon uptake and release, determining whether croplands function as net carbon sinks or sources (Hollinger et al., 2005; Liu et al., 2022; Li et al., 2022). Accurately quantifying carbon fluxes and net carbon balance under specific agricultural management is therefore essential to reduce uncertainties in the terrestrial carbon budget.

Cotton production in China is highly concentrated in Xinjiang, which accounts for more than 90% of national output and planted area. Drip-irrigated and plastic-mulched (DIPM) is the dominant management practice in this region, widely adopted for its water-saving and yield-enhancing benefits. Numerous field experiments have shown that DIPM improves soil hydrothermal conditions, enhances canopy development and photosynthetic capacity, and increases yield (Li et al., 2011; Zhang et al., 2022, Zhang et al., 2023). Cotton straw is typically returned to the field after harvest to improve soil fertility and yield (Chen et al., 2023). Yet, its net carbon benefit depends on carbon partitioning between yield and residue, a trade-off that remains poorly understood (Yin et al., 2025). This partitioning, in turn, is fundamentally governed by biotic processes such as canopy development, biomass accumulation, and root-shoot carbon allocation, which determine both the magnitude of carbon fixation and its distribution between harvestable products and residue. Therefore, understanding how biotic factors regulate carbon fluxes across different growth stages is essential for assessing the net carbon benefit of straw return and, more broadly, the carbon sink potential of DIPM cotton systems.

Most existing studies have relied on traditional methods, such as leaf-level photosynthesis measurements, biomass sampling, and chamber-based soil respiration, which provided plot-scale insights but cannot capture ecosystem-scale CO_2_ exchange or quantify the net carbon balance of the entire agroecosystem. Eddy covariance (EC) techniques overcome these limitations by providing continuous, half-hourly measurements of net ecosystem exchange (NEE), gross primary production (GPP), and ecosystem respiration (R_eco_) over multiple growing seasons. EC has been widely deployed across various cropping systems worldwide, including staple crops, cash crops, and crop rotations (Grant et al., 2007; Wang et al., 2015; Tao et al., 2022). In China, long-term EC observations have substantially advanced understanding of carbon and water fluxes across agroecosystems, from the North China Plain to the arid river basins of the northwest (Zhang et al., 2013; Yue et al., 2023).

For cotton systems specifically, EC studies have been conducted in major cotton-producing regions worldwide. Globally, these studies have shown that cotton can act as a net carbon sink during the growing season, though the magnitude varies considerably with climate, irrigation, and management practices (Fong et al., 2020; Menefee et al., 2020; Chakraborty et al., 2022). Beyond EC-based flux measurements, complementary approaches have also advanced cotton carbon budget research, including process-based modeling and model-data fusion (Qin et al., 2025). In Xinjiang DIPM cotton systems, EC studies have reported growing-season NEE ranging from -305 to -479 g C m⁻² (Bai et al., 2015; Li et al., 2018). However, these earlier studies, including the four-year record of Bai et al. (2015) at the same site, did not examine stage-specific shifts in biotic versus abiotic controls, nor did they integrate EC-derived net uptake with straw return to estimate the net ecosystem carbon budget (NECB). The present study extends this previous work by providing a longer observational record (ten growing seasons), employing a consistent GPP/R_eco_ partitioning method, and explicitly incorporating straw return into the NECB accounting framework. Ming et al. (2021) further estimated the NECB of DIPM cotton in southern Xinjiang, reporting a weak carbon source after accounting for harvest export. Furthermore, while straw return is widely adopted in Xinjiang cotton production, its contribution to NECB has rarely been quantified through direct integration with EC measurements.

To address these gaps, this study analyzes ten growing seasons (2009-2013, 2016-2019, and 2022) of EC, meteorological, crop growth, and management data from a DIPM cotton system in northern Xinjiang. Understanding the physiological responses of GPP to key climatic drivers is essential for attributing carbon flux variability to biotic versus abiotic factors. We hypothesized that the dominant drivers of carbon fluxes shift from biotic to abiotic factors across cotton developmental stages, and that straw return plays a critical role in sustaining the NECB of the DIPM cotton system. The specific objectives are: (1) to characterize seasonal and interannual carbon fluxes; (2) to quantify the responses of daytime GPP to light, vapor pressure deficit (VPD), and air temperature (T_a_); (3) to determine the stage-specific contributions of biotic versus abiotic factors using random forest modeling; and (4) to evaluate the NECB by integrating EC-derived net ecosystem production with management carbon inputs and outputs, with particular attention to straw return.

## 2 Materials and method

### 2.1 Study sites

**Figure 1:**
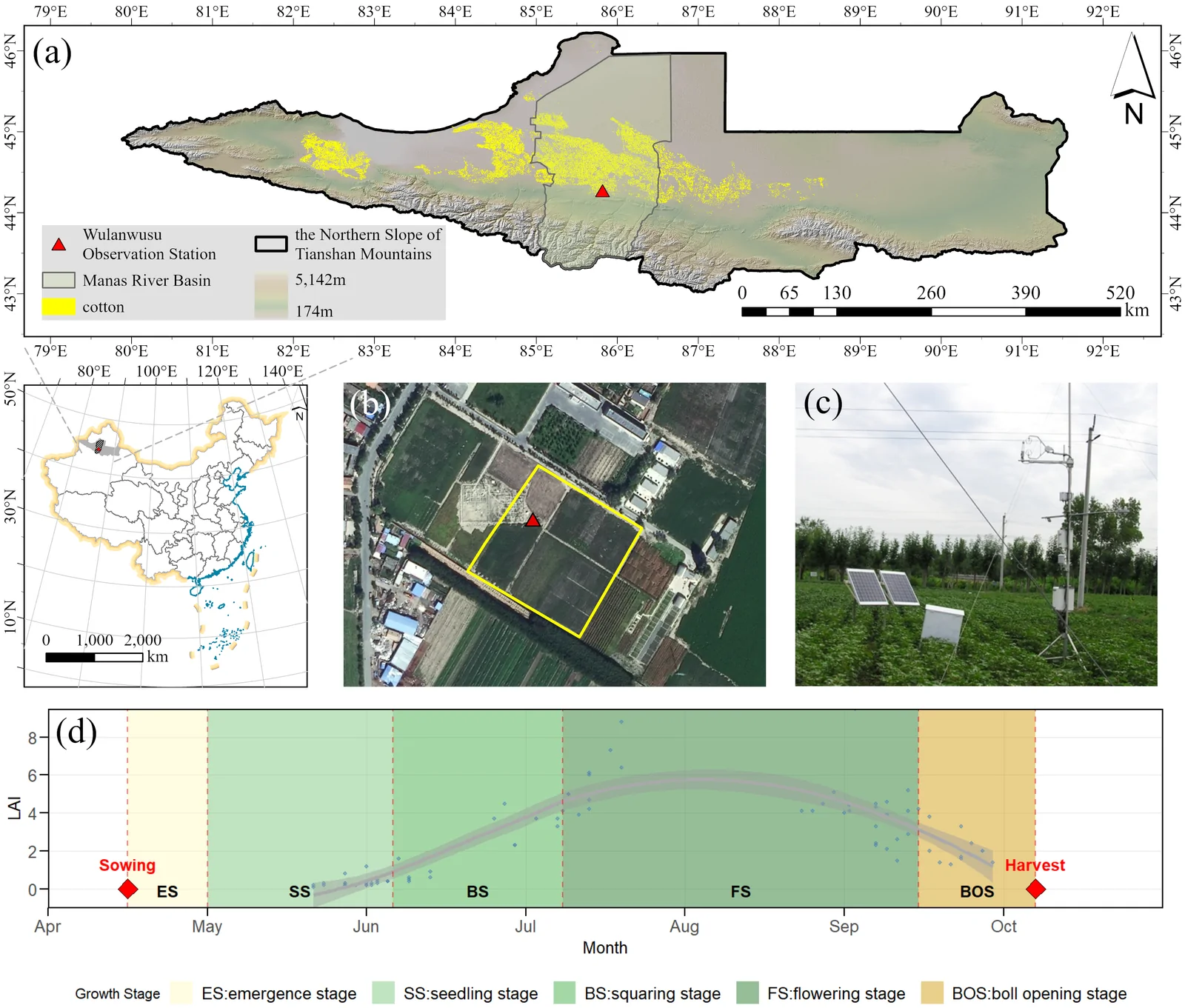
**(a)** Location of the study field in main cotton-growing region of northern Xinjiang, **(b)** Satellite image showing the cotton field (yellow outline) and EC tower location (red triangle), **(c)** the photo of EC flux tower, and **(d)** the phenological curves of cotton across five growth stages: ES (emergence), SS (seedling), BS (squaring), FS (flowering), and BOS (boll opening). Colored lines represent individual growing seasons (2009-2013, 2016-2019, 2022).

The experiment was conducted at the Wulanwusu Agrometeorological Experiment Station (WLWS_AES), (44.30°N, 85.87°E, 469 m) in the Manas Oasis on the northern slope of the Tianshan Mountains, Xinjiang, Northwest China (**Figures 1a–c**). This site has a temperate arid continental climate, with an average annual temperature of 7.62 °C, precipitation of 215.2 mm, evaporation of 2146.0 mm, and sunshine duration of 2832 h. The soil is slightly alkaline sandy loam, with a bulk density of 1.32 g cm⁻³, field capacity of 23.76%, and wilting coefficient of 5.76%.

Cotton (*Gossypium hirsutum L.*) is grown under a continuous single-cropping system, and the dominant cultivar is “Xinluzao”. Cotton is typically established using flat planting with plastic mulching and drip irrigation. Sowing occurs in late April (around DOY 105) at a seeding rate of 44.36 ± 1.86 kg ha^⁻¹^ and a depth of 2 cm, with harvested in early October (around DOY 276). After harvest, most residues are mechanically chopped into the 0–30 cm soil layer. During the growing season, irrigation is applied 7–9 times, totaling 427.07 ± 134.4 mm, with nitrogen fertilizer (17.29 g N m⁻²) applied through drip fertigation in 4–5 applications. At maturity, plant height averages 72.56 ± 5.68 cm, planting density 24.09 ± 2.54 plants m⁻², and seed-cotton yield 4857.00 ± 648.82 kg ha^-1^ (**Supplementary Table 1**).

The WLWS_AES maintains long-term agrometeorological observations (1994–2023) of cotton growth, yield, management practices, phenology, and soil moisture, with EC-based carbon flux measurements available for 2009–2013, 2016–2019, and 2022. Based on these records, the cotton growing season is divided into five stages: emergence stage (ES), seedling stage (SS), squaring stage (BS), flowering stage (FS), and boll opening stage (BOS) (**Figure 1d**). The full season lasts approximately 172 days: 14 days for ES, 36 days for SS, 31 days for BS, 68 days for FS, and 22 days for BOS (**Supplementary Table 2**).

### 2.2 Eddy covariance and meteorological measurements

At WLWS_AES, carbon fluxes in DIPM cotton system were measured by an open-path eddy covariance (EC) system, which consists of an open-path and fast-response infrared gas analyzer (LI-7500, Li-Cor Inc., USA) for measuring CO_2_ and H_2_O densities, and a three-dimensional sonic anemometer (CSAT 3, Campbell Scientific Inc., USA) to measure the turbulent fluctuations in wind components and sonic (virtual) temperature. The EC tower, installed in August 2008 in the center of a homogeneous cropland field, was equipped with sensors mounted 4.0 m above the ground. High-frequency EC signals were sampled at 10 Hz and recorded with a data logger (CR3000, Campbell Scientific Inc, Logan, UT, USA).

An automatic meteorological system was installed about 5 m from the EC system. Air temperature and humidity were measured with a HMP45D probe (HMP45D, Vaisala, Inc., Helsinki, Finland) at 2.0 m above ground level. The atmospheric pressure (PTB220, Vaisala, Inc., Helsinki, Finland), and wind speed and direction (EL15-1A wind speed sensor and the EL15-2D wind direction sensor, Zhonghuan TIG, Tianjin, China) were also set at 2.0 m above ground level. The net radiation (R_net_) was measured with a radiometer (CNR-1, Kipp & Zonen, Delft, Netherlands) at 3.0 m above ground level. Precipitation was measured using a weighing-type precipitation sensor (Model DSC1; Jiangsu Radio Science Research Institute Co., Ltd. Wuxi City, China). The soil heat flux (G) was measured with two soil-heat-flux plates (HFP01, Hukseflux, Delft, the Netherlands) at 80-mm depth below the ground surface. Soil temperature and soil water content (SWC) were measured using a TCAV probe (Campbell Scientific Inc., Logan, UT, USA) and TDR probe (CS615-L, TDR, Campbell Scientific), at 20 and 40 mm below the soil surface. Meteorological data were recorded every hour using a datalogger (Model CR1000X, Campbell Scientific Inc., USA).

### 2.3 Eddy covariance data processing

The eddy covariance data in this study were processed through four steps as follows: (1) raw data collection, (2) data preprocessing, (3) quality control, and (4) gap-filling. The high-frequency (10 Hz) raw data were initially processed into 30-minute fluxes using EddyPro software (v7.0.6, LI-COR, Lincoln, NE, USA) with double coordinate rotation and WPL corrections. The raw 30-minute fluxes data underwent a multi-step quality control (QC) procedure. Following the standard procedures of ChinaFLUX (Yu et al., 2006), nighttime flux data measured under low turbulence conditions were filtered using a friction velocity (u*) threshold. The threshold was determined using the moving point test (Reichstein et al., 2005), with a site-specific u* threshold of 0.15 m s⁻¹. Data with u* below this threshold were removed prior to gap-filling. Firstly, individual data points with values outside the range of -40 to 30 μmol CO_2_ m⁻² s⁻¹ were removed as spurious outliers. This range was determined based on the distribution of our flux data and is consistent with the magnitudes reported for the same site (Bai et al., 2015). Secondly, a 7-day moving window was applied to detect statistical outliers (spike), with any data point deviating from the window mean by more than three standard deviations (|X_i_ − X̄| ≥ 3σ) flagged and excluded (Vickers and Mahrt, 1997).

Due to frequent missing data segments in the WLWS_AES fluxes time series, a gap-filling method based on Look-Up Tables (LUTs) was employed, as widely used in ecosystem flux studies (Falge et al., 2001). It leveraged the strong annual periodicity and relative stability of ecological variables during the same time periods across different years. Specifically, for constructing the LUTs, a 7-day moving window was used across multiple years to balance interpolation stability with the influence of external variables, following the approach of Baldocchi et al. (2001). Considering diurnal variability, an additional hourly sliding window of ±1 hour was used to capture correlations at sub-daily timescales. The control variables that most strongly influence the target flux variable (e.g., NEE) were selected, such as incoming solar radiation (R_g_), T_a_, and relative humidity (RH). The LUT bin sizes (tolerance ranges) were set as follows: ± 50 W m⁻² for R_g_, ± 2 °C for T_a_, and ±10% for RH, based on Wutzler et al. (2018). Fluxes values were then gap-filled by replacing missing values with the mean flux of the selected records under similar meteorological conditions within the specified windows and constraints. This combined approach, integrating temporal window expansion with multi-variable constraints, provides a robust and ecologically relevant framework for interpolating missing flux data at WLWS_AES.

After gap filling, NEE was partitioned into GPP and R_eco_ using the daytime-based method implemented in the REddyProc package in R (V 4.3.3) (Wutzler et al., 2018), following the algorithm of Lasslop et al. (2010). In this method, daytime NEE data are used to simultaneously fit a light-response curve (rectangular hyperbola) for GPP and a temperature-dependent respiration function for R_eco_. GPP was then calculated as using Equation 1:

(1) $$\text{GPP}=R_{eco}-\text{NEE}$$

where GPP and R_eco_ are defined as positive (carbon gain and carbon loss, respectively). Negative NEE indicates net CO_2_ uptake by the ecosystem (carbon sink), whereas positive NEE indicates net CO_2_ release to the atmosphere (carbon source).

### 2.4 Crop sampling and measurements

LAI and aboveground dry biomass (ABS) were measured at five phenological stages: seedling (including the five-leaf stage), squaring, flowering (including full-blooming), boll development, and boll-opening. Sampling was conducted within a 0.3 km × 0.3 km plot located 2 km west of the EC tower. Four sampling locations were established at the four corners of the plot. At each location, 10 cotton plants were randomly selected (40 plants per campaign) and the aboveground parts were harvested. For LAI estimation, one large, one medium and one small leaf were collected from each plant; the maximum length and height of leaf were measured, and individual LAI was calculated as 0.75 × length × width (length and width in cm). Harvested plants were separated into leaves, petioles, stems (branches) and bolls, and fresh weight was recorded for each component. Samples were then green-killing at 100-105 °C for 30 min, and then dried at 70-80 °C for 8–10 hours to constant mass to determine dry weight. During flowering and boll-opening stages, the height of the selected plants (40 plants in total) was measured from the soil surface to the top of the main stem and the average plant height was calculated. Meanwhile, the number of cotton plants within a 1-m row length was counted in the direction of vertical strips in each of the four sampling points and repeated four times, and then the average value was taken as the plant density of the sampling point. Finally, plant-based LAI (m^2^ plant^-1^) and ABS (g plant^-1^) were converted to area-based LAI (m^2^ m^-2^) and ABS (g m^-2^) by multiplying by plant density.

In addition to the intensive sampling campaigns described above, long-term biomass records (1994-2023) were obtained from the WLWS_AES agrometeorological observation program. ABS was sampled annually at key phenological stages following the same protocol described in this section, and the data were used to derive the long-term trends in NPP, yield, and biomass allocation presented in Section 3.5 and discussed in Section 4.3.

### 2.5 Carbon budget calculation

Field surveys indicated that cotton at WLWS_AES was managed without residue burning, and approximately 85% of the straw was mechanically chopped and returned to the field after harvest. To quantify the net carbon balance of the DIPM cotton system, the NECB was calculated by combining growing-season NEP derived from EC measurements with lateral carbon fluxes associated with harvest export and straw return, following the framework established for agricultural ecosystems (Béziat et al., 2009; Ming et al., 2021; Liu et al., 2019).

Following the micrometeorological sign convention, NEE is defined as positive when CO_2_ is released from the ecosystem to the atmosphere and negative when CO_2_ is taken up by the ecosystem. NEP is defined as the negative of cumulative NEE during the growing season, thus a positive NEP indicates net carbon uptake using Equation 2:

(2) $$\text{NEP}=-\sum_{GS}NEE$$

WhereΣ_GS_ denotes summation over the growing season. Units are g C m⁻².

With carbon inputs to the field defined as positive and carbon exports defined as negative, NECB was calculated as using Equation 3 (Béziat et al., 2009; Ming et al., 2021; Liu et al., 2019):

(3) $$\text{NECB}=\text{NEP}+C_{seed}+C_{straw}-C_{harvest}$$

Where *C_seed_* (g C m^-2^) is carbon input from sowing seeds, *C_straw_* (g C m^-2^) is carbon inputs by straw return, and *C_harvest_* (g C m^-2^) is carbon removed from seed-cotton harvest.

Carbon export through seed-cotton harvest was estimated as using Equation 4 (Ming et al., 2021; Yue et al., 2023):

(4) $$C_{harvest}=(1-W_{c})\times Y\times f_{c}\times 1000$$

Where *W_c_* is water percentage of seed-cotton harvest (0.12), *Y* (kg m^-2^) is seed-cotton yield, and *f_c_* is the carbon fraction (0.45). The factor 1000 converts kg to g.

Carbon input from sowing seeds was estimated as using Equation 5 (Liu et al., 2019):

(5) $$C_{seed}=(1-W_{s})\times M_{seed}\times f_{c}\times 1000$$

Where *W_s_* is the water percentage of sowing seeds (0.10), *M_seed_* is the sowing seeds mass (kg m^-2^), and *f_c_* is the carbon fraction (0.45).

Carbon input from straw return was calculated as using Equation 6 (Ming et al., 2021):

(6) $$C_{straw}=(NPP-C_{harvest})\times f_{R}$$

Where *f_R_* is the proportion of straw returned to the soil (0.85). Here, $NPP-C_{harvest}$ represents the total carbon in crop residues (stems, leaves, boll shells and roots) before accounting for the fraction actually returned to the field.

Net primary productivity (NPP) represents the total carbon fixed by plants during the growing season. It was estimated as the sum of aboveground and belowground biomass carbon. It was calculated using Equation 7 (Ming et al., 2021):

(7) $$\text{NPP}=(ABS_{stem}+ABS_{leaves}+ABS_{bolls})\times (1+R_{r\_s})\times f_{c}$$

Where *ABS_stem_*, *ABS_leaves_*, *ABS_bolls_* are the aboveground dry biomass of stems, leaves and bolls (g m⁻²), and *R_r_s_* is the root shoot ratio (0.11), and *f_c_* is the carbon fraction (0.45). The root shoot ratio was derived from destructive root sampling conducted in 2012 and 2013 at the WLWS_AES site.

### 2.6 Response functions of GPP to environmental factors

The functional forms of GPP to *R_net_*, *VPD* and *T_a_* in this study are taken from Whitley et al. (2009, 2011). The boundary-line method was used, which fits the upper envelope of the GPP response to each environmental variable. By filtering data to retain only those points where non-target factors were near optimal, this approach captures the potential response ceiling rather than the average response, and thus reveals nonlinear saturation patterns often obscured in conventional regressions.

*R_net_* is the primary energy source for photosynthesis. GPP increases rapidly with *R_net_* under non-limiting water and nutrient conditions, but saturates under light-saturating conditions when other factors become limiting. This pattern is described by a rectangular hyperbola function (Equation 8):

(8) $$\text{Y}=\text{a }\times \;\frac{\text{X}}{\text{X}+\text{b}}$$

where, *a* is the light-saturation *GPP* (*GPP_max_*, μmol CO_2_ m⁻² s⁻¹), *X* is the *R_net_* (W m⁻²), and *b* (W m⁻²)) is a fitted parameter controlling the initial slope (the rate of increase in GPP with *R_net_* at low light). A larger *b* value indicates a slower initial response of GPP to increasing *R_net_*, implying lower photosynthetic efficiency under light-limited conditions.

VPD and T_a_ influence GPP through stomatal conductance, with high VPD inducing stomatal closure and suboptimal temperatures suppressing enzymatic activity. The response typically increases with rising VPD or T_a_ up to an optimum, then declines beyond that threshold. This pattern is described by a modified exponential function (Equation 9):

(9) $$\text{Y}=\text{c}\times \text{exp}\{\frac{-{\text{k}}_{1}\times {\left(\text{X}-{\text{k}}_{3}\right)}^{2}}{\text{X}+{\text{k}}_{2}}\}$$

where, *c* is the maximum *GPP* (*GPP_max_*, μmol CO_2_ m⁻² s⁻¹), *X* is either *T_a_* (°C) or *VPD* (kPa), *K_1_* and *K_2_* are the shape parameters controlling steepness and asymmetry of the curve, and *K_3_* describes the optimal *T_a_* or *VPD* where *GPP* peaks. Note that when the range of *X* is limited, the fitted *K_1_* and *K_2_* can be sensitive to data variability, while *K_3_* is generally more stable.

### 2.7 Statistical methods

The random forest (RF) model is used to assess the importance of main driving factors (i.e., LAI, ABS, R_net_, VPD, T_a_, soil temperature (T_s_), precipitation (P), and SWC for NEE, GPP, and R_eco_ across the five growth stages. In the RF model, 70% of the original dataset is allocated as the training set while the remaining 30% is used as the validation set to evaluate the model’s overall error and the importance of each variable. The coefficient of determination (R²) and root mean square error (RMSE) are obtained through 5-fold cross-validation to assess the model’s stability and generalizability (Yue et al., 2023). The RF model is constructed using the “randomForest” package in R, while the “rfPermute” package in R is employed to evaluate the importance of each driving factor on NEE, GPP, and R_eco_ during growth stages. Before building the RF model, the augmented Dickey-Fuller test is applied to examine the stationarity of the dataset (i.e., NEE, GPP, R_eco_, and driving factors), and first-order differencing is performed on data exhibiting temporal autocorrelation. Multicollinearity among predictor variables is assessed using variance inflation factor (VIF). As is common under field conditions, T_a_ and T_s_ show moderate to strong collinearity, with VIF values up to 20 across different growth stages, while all other predictors have VIF values below 5. Because random forest is robust to correlated predictors and does not rely on linear assumptions, all variables are retained in the model; however, the importance of T_a_ and T_s_ is interpreted jointly. The optimal parameter values are selected and tuned using cross-validation. In this study, the RF model is set with 1000 decision trees (ntree), 8 random subsets of predictors (mtry), and nodesize of 5 (nodesize). Linear regression analysis was used to evaluate the interannual trends of carbon budget components (NEP, C_harvest_, C_straw_, and NECB). The slope of the linear regression indicated the rate of change per year, and the coefficient of determination (R²) was used to assess the goodness of fit. Variable importance in RF model reflects the relative contribution of each predictor to prediction accuracy (measured as %IncMSE). As with any observational study, these importance values indicate statistical association rather than causation, and should be interpreted in the context of the collinearity and potential endogeneity inherent in field data.

## 3 Results

### 3.1 Meteorological factors and crop development

**Figure 2:**
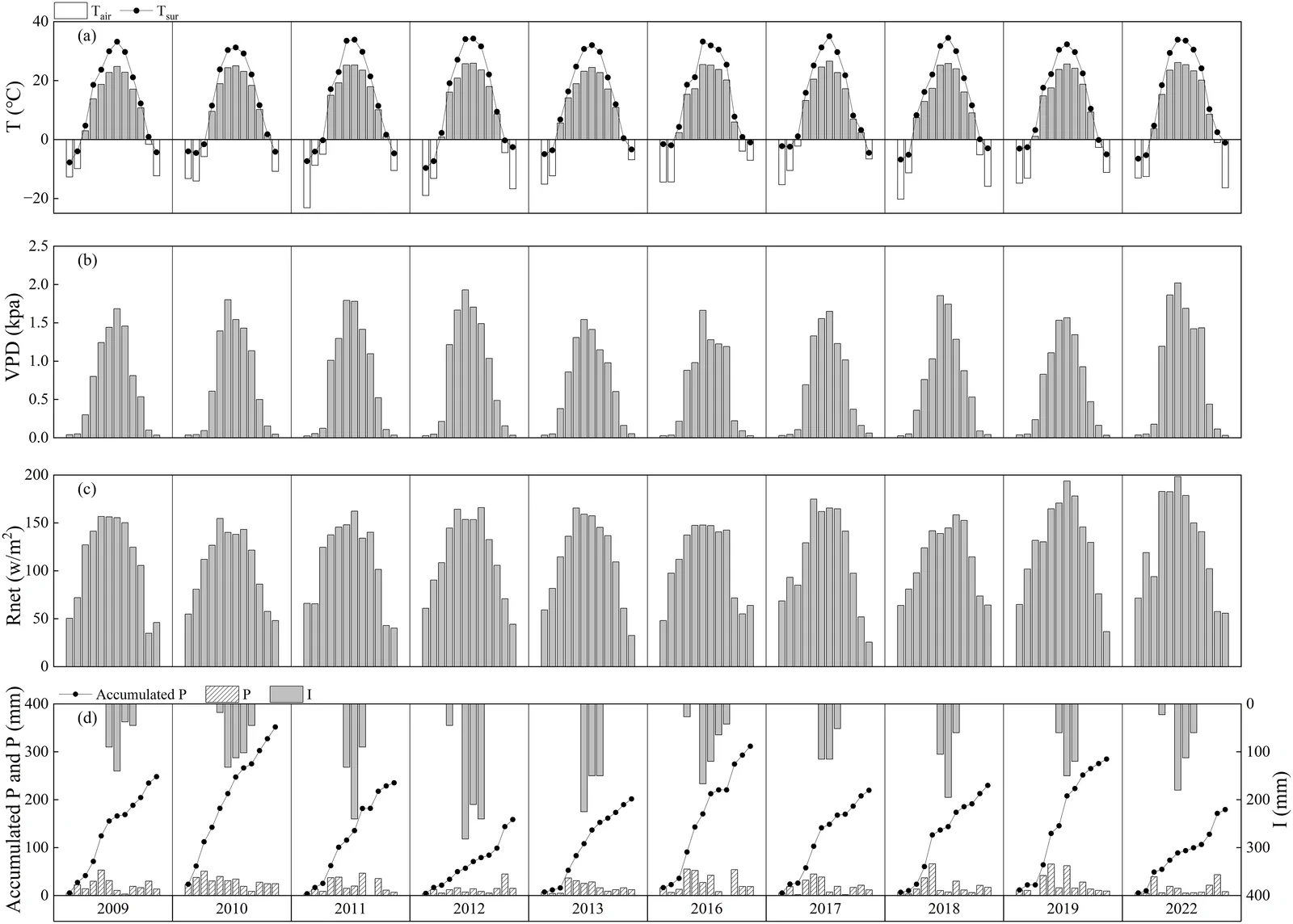
Dynamics of monthly meteorological factors at WLWS_AES during 2009-2013, 2016-2019, and 2022 (excluding 2014–2015 and 2020–2021 due to instrument system malfunctions that resulted in incomplete flux records): **(a)** air temperature (T_a_) and surface temperature (T_s_), **(b)** vapor pressure deficit (VPD), **(c)** net solar radiation (R_net_), **(d)** accumulated precipitation (Accumulated P), monthly precipitation (P) and irrigation amount (I).

Meteorological conditions at the DIPM cotton system showed consistent seasonal variations (**Figure 2**). The T_a_, T_s_, VPD, and R_net_ all displayed a unimodal seasonal pattern, with minimum values in January (T_a_: -12.7 ± 4.3 °C; T_s_: -7.8 ± 2.8 °C; VPD: 0.03 ± 0.01 kPa; R_net_: 60.3 ± 7.6 W m^-^²) and maximum values in July (T_a_: 25.3 ± 1.3 °C; T_s_: 33.2 ± 1.3 °C; VPD: 1.68 ± 0.17 kPa; R_net_: 165.4 ± 16.3 W m^-^²). Precipitation also showed distinct seasonality, with the lowest monthly mean in winter (December-February: 6.3 ± 6.1 mm month^-1^) and the highest in summer (June-August: 21.3 ± 14.8 mm month^-1^) (**Figure 2d**). Consistent with this seasonal pattern, about 62% of the annual irrigation was applied during the summer months.

At the interannual scale, mean annual T_a_ varied from 7.08 °C in 2018 to 8.57 °C in 2022, while mean annual T_s_ varied from 12.26 °C in 2010 to 14.52 °C in 2022 (**Figure 2a**). Annual precipitation showed greater variability, ranging from 158.5 mm in 2012 to 351.8 mm in 2010. Growing-season precipitation followed a similar pattern, with the lowest totals in 2012 (62.5 mm) and 2022 (57.4 mm) and the highest in 2019 (229.3 mm) (**Figure 2d**). Annual mean VPD ranged from 0.65 kPa in 2016 to 0.87 kPa in 2022 (**Figure 2b**), while R_net_ increased in recent years, peaking at 131.0 W m⁻² in 2022 (**Figure 2c**). Annual irrigation amounts varied substantially and were negatively correlated with precipitation. The highest irrigation (777.0 mm in 2012) coincided with the lowest annual precipitation (**Figure 2d**). Consequently, relatively dry years (e.g., 2012 and 2022) were characterized by low precipitation and higher VPD alongside greater irrigation inputs, while wetter years (e.g., 2010 and 2016) had higher precipitation and lower VPD, with irrigation amounts closer to the multi-year mean.

**Figure 3:**
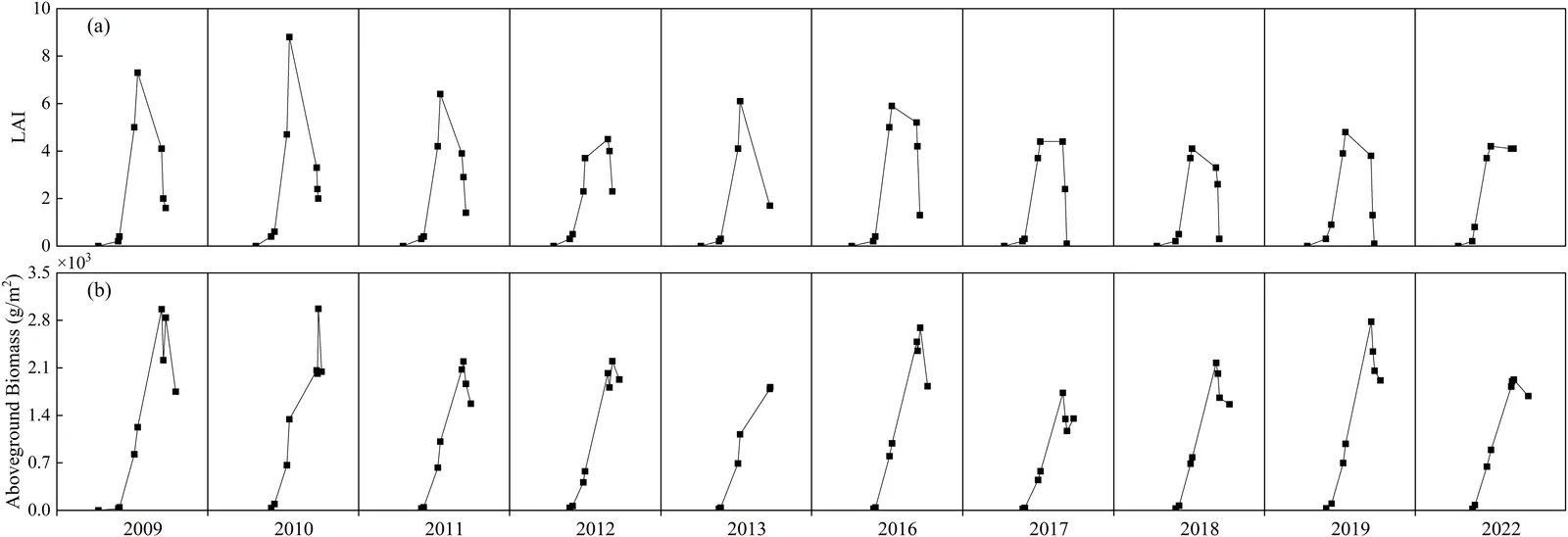
Seasonal variations of **(a)** leaf area index (LAI, m^2^ m^-2^) and **(b)** aboveground dry biomass (ABS, g m^-2^) at WLWS_AES during the growing seasons of 2009-2013, 2016-2019, and 2022.

The seasonal dynamics of LAI and ABS in the DIPM cotton system closely followed cotton phenological development, but showed pronounced interannual variability (**Figure 3**). LAI remained near zero at SS (April-May), increased rapidly after BS (June), peaked (4.0-8.8) at FS (mid-to-late July), and then declined sharply during BOS (September) (**Figure 3a**). In contrast, ABS accumulated slowly during SS (< 30 g m⁻²), grew rapidly from BS to FS (June-July; 400–1300 g m⁻²), and reached its maximum at BOS (August-September; 1500–3000 g m⁻²) (**Figure 3b**). Notably, ABS remained high at BOS even though LAI decreased which indicating continued biomass accumulation and assimilate allocation to bolls later in the period. Across years, the date of peak LAI shifted by about one month (mid-July to late August). In the dry year (2012), peak LAI occurred later (August) and was relatively low (4.5), whereas in the wetter year (2010) the maximum LAI (8.8) occurred earlier (18 July). Peak ABS also varied markedly among years (multi-year mean: 1745.13 ± 196.04 g m⁻²), with the highest value in 2010 (2043.10 g m⁻²) and the lowest in 2017 (1352.30 g m⁻²).

### 3.2 Seasonal and interannual variations in carbon fluxes of DIPM cotton system

#### 3.2.1 Monthly dynamics of carbon fluxes

**Figure 4:**
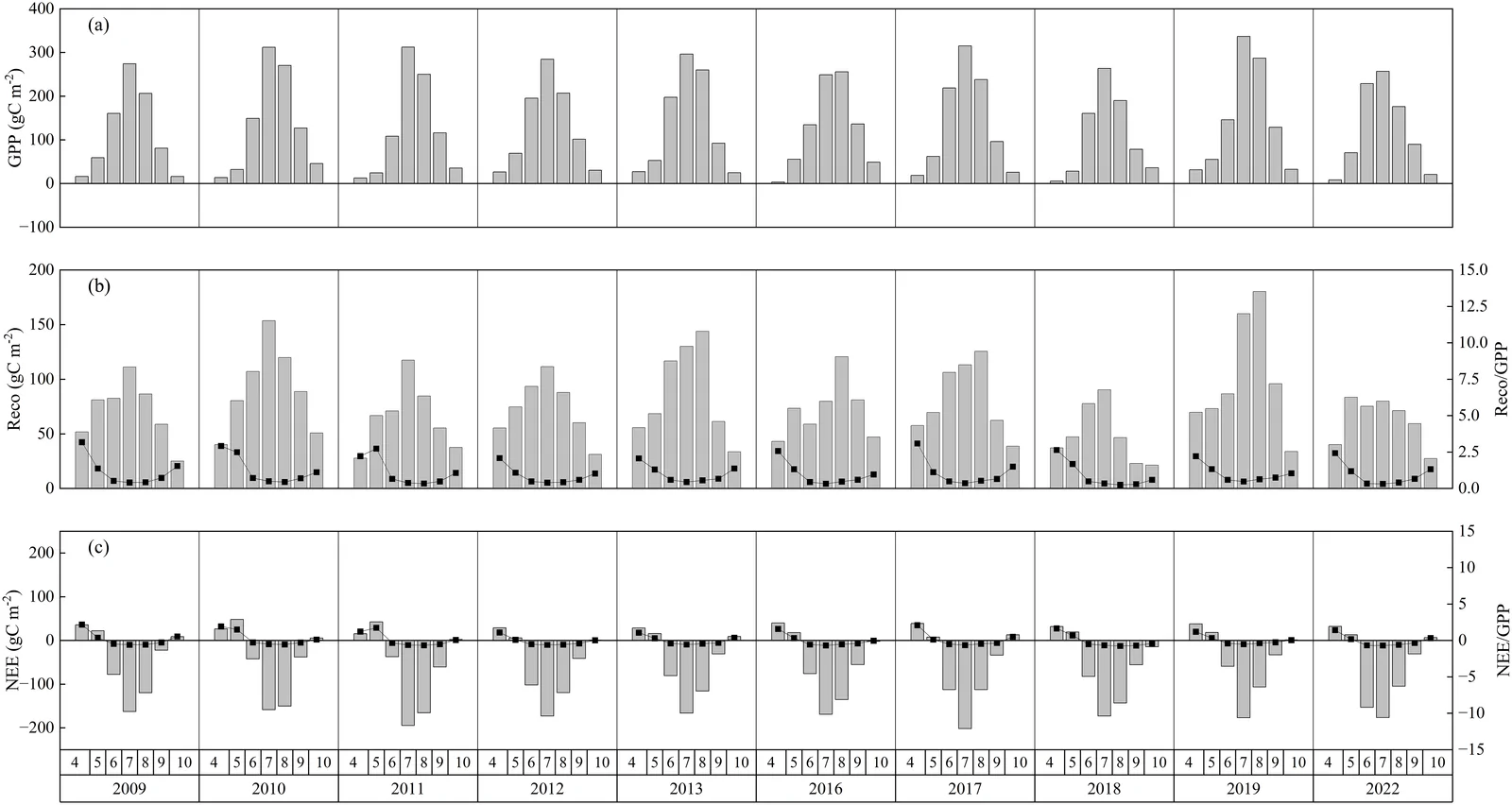
Monthly dynamics of **(a)** gross primary productivity (GPP), **(b)** ecosystem respiration (R_eco_) and **(c)** net ecosystem exchange (NEE) of the DIPM cotton system from April to October in 2009-2013, 2016-2019, and 2022. Dashed lines represent the R_eco_/GPP and NEE/GPP ratio.

Monthly cumulative carbon fluxes (g C m⁻²) in the DIPM cotton system showed strong seasonality (**Figure 4**). NEE was positive in the early season (April-May), then shifted to negative values during vigorous growth (June-September), and returned to positive values in October (**Figure 4c**). Over the same period, GPP increased sharply into mid-season and peaked in July-August (~200–300 g C m⁻²; **Figure 4a**), whereas R_eco_ varied more modestly, remaining ~80–120 g C m⁻² in mid-season and declining to <60 g C m⁻² at the beginning and end of the growing season (**Figure 4b**).

Key months revealed the extent of the seasonal source-sink transition. In April, GPP was minimal (16.33 ± 9.61 g C m⁻²) and much lower than R_eco_ (47.83 ± 12.32 g C m⁻²), resulting in positive NEE (31.47 ± 7.35 g C m⁻²). By June, GPP increased to 169.85 ± 38.72 g C m⁻², exceeding R_eco_ (87.53 ± 18.23 g C m⁻²), and NEE became negative (-82.28 ± 34.26 g C m⁻²). Net CO_2_ uptake peaked in July, when GPP and R_eco_ reached their seasonal maxima (289.94 ± 29.08 and 114.71 ± 27.59 g C m⁻², respectively) and NEE was most negative (-175.24 ± 13.62 g C m⁻²). By October, GPP declined to 31.61 ± 10.37 g C m⁻² and approached R_eco_ (34.65 ± 9.29 g C m⁻²), with NEE close to zero (3.02 ± 7.67 g C m⁻²) (**Figure 4**). NEE in July contributed approximately 60% to the seasonal cumulative NEE.

Carbon fluxes partitioning ratios showed consistent seasonal trends (**Figures 4b, c**). The NEE/GPP ratio was positive in the early season (April: 1.93; May: 0.41), became strongly negative during peak uptake (July: -0.60), and approached zero by October (0.10). The R_eco_/GPP ratio was highest in April (2.93), lowest during peak uptake (July: 0.40), and increased again toward harvest (October: 1.10).

#### 3.2.2 Growth-stage differences in carbon fluxes

**Figure 5:**
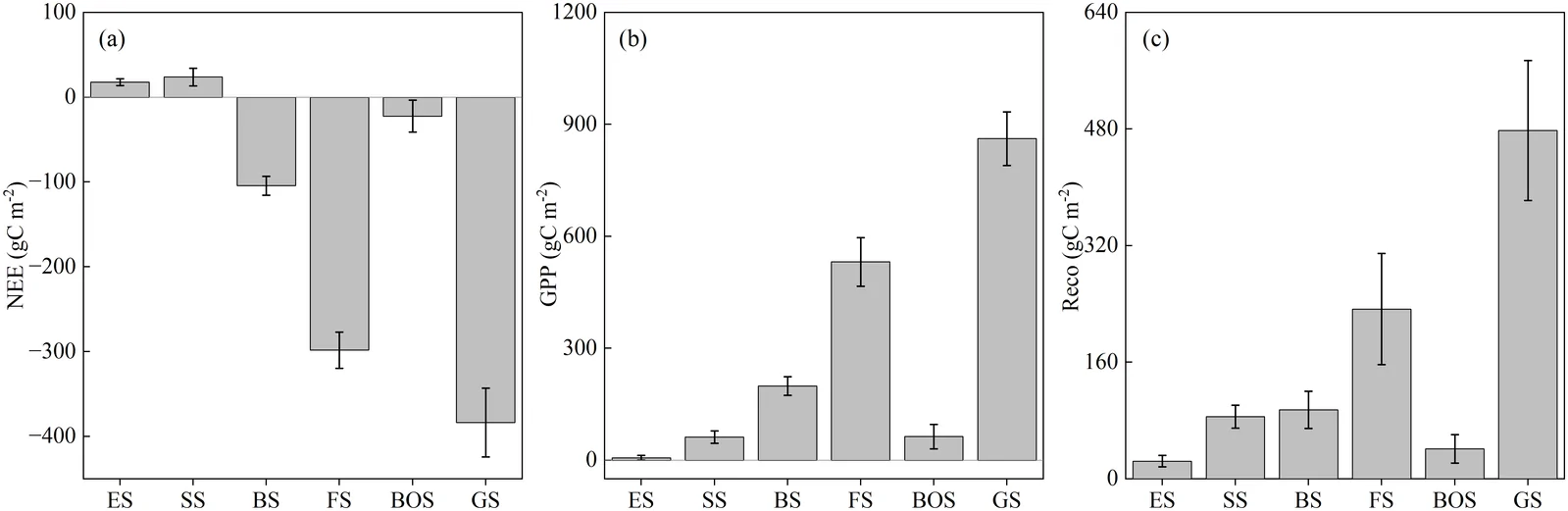
Multi-year mean (2009-2013, 2016-2019, 2022) of **(a)** GPP, **(b)** R_eco_ and **(c)** NEE across five growth stages: ES, SS, BS, FS, and BOS. Error bars represent standard deviation (n = 10 growing seasons).

All flux values reported in this section are cumulative totals for the growing season only (from sowing to harvest, approximately April-October). Carbon flux partitioning varied markedly among growth stages (**Figure 5**), with cumulative fluxes calculated for each stage (g C m⁻²). During vegetative development (ES and SS), R_eco_ exceeded GPP (R_eco_/GPP = 2.93) and NEE was positive (41.42 ± 7.29 g C m⁻²). During reproductive development (BS-BOS), the R_eco_/GPP ratio decreased to 0.40 and NEE became strongly negative (-425.03 ± 17.13 g C m⁻²), indicating net CO_2_ uptake. The FS accounted for the largest carbon fluxes, with GPP and R_eco_ of 531.27 ± 65.51 and 232.96 ± 76.45 g C m⁻², contributing 61.66% of seasonal GPP and 48.73% of seasonal R_eco_, respectively. NEE was most negative at FS (-298.33 ± 21.47 g C m⁻²), accounting for 77.77% of total seasonal carbon sequestration, compared with BS (-104.33 ± 11.13 g C m⁻²) and BOS (-22.37 g C m⁻²) (**Figure 5**). Across years, reproductive stages (BS-BOS) contributed ~92% of total GPP, with BS and FS contributing the most to seasonal net CO_2_ uptake.

#### 3.2.3 Interannual variability in growing-season carbon fluxes

**Figure 6:**
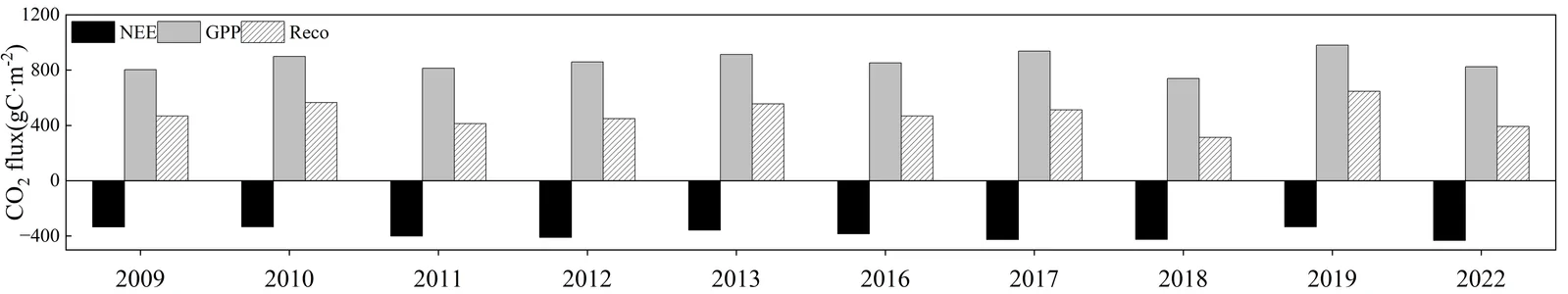
Growing-season cumulative NEE, R_eco_, and GPP of the DIPM cotton system in 2009-2013, 2016-2019, and 2022.

Across the observed growing seasons (2009-2013, 2016-2019, and 2022), the DIPM cotton system consistently functioned as a net CO_2_ sink during the growing season (NEE < 0), although sink strength varied substantially among years (**Figure 6**). Seasonal cumulative NEE (integrated over the growing season) ranged between -334.07 and -431.62 g C m⁻², with the strongest sink in 2022 (-431.62 g C m⁻²) and the weakest in 2010 (-334.07 g C m⁻²). The difference between the two extreme years was 97.55 g C m⁻², with a coefficient of variation of 14% for seasonal NEE across the ten growing seasons. Seasonal cumulative GPP exhibited pronounced interannual variability, ranging from 738.68 to 980.20 g C m⁻², with a difference of 241.52 g C m⁻² and a CV of 10%. The highest GPP occurred in 2019, while the lowest was recorded in 2018. Similarly, seasonal cumulative R_eco_ followed a similar pattern, reaching a maximum in 2019 (646.54 g C m⁻²) and a minimum in 2018 (312.79 g C m⁻²), with a difference of 333.75 g C m⁻² and a CV of 21%.

Seasonal NEE/GPP ratios ranged from -0.34 to -0.58, indicating that ~34-58% of assimilated carbon corresponded to net CO_2_ uptake during the growing season. NEE/GPP ratio was highest in 2018 (-0.58) and lowest in 2019 (-0.34). Consistently, the seasonal R_eco_/GPP ratio ranged between 0.42-0.66, showing lowest respiratory carbon loss in 2018 (0.42) and highest in 2019 (0.66). Overall, these results indicate that the carbon sink strength of the DIPM cotton system was jointly regulated by interannual variability in photosynthetic carbon uptake and ecosystem respiration. Enhanced carbon sequestration occurred in 2017–2018 and 2022, whereas weaker sink strength was observed in years such as 2010 and 2019, when higher respiratory losses reduced net carbon gains.

### 3.3 Responses of daytime GPP to climate variables at critical growth stages

**Figure 7:**
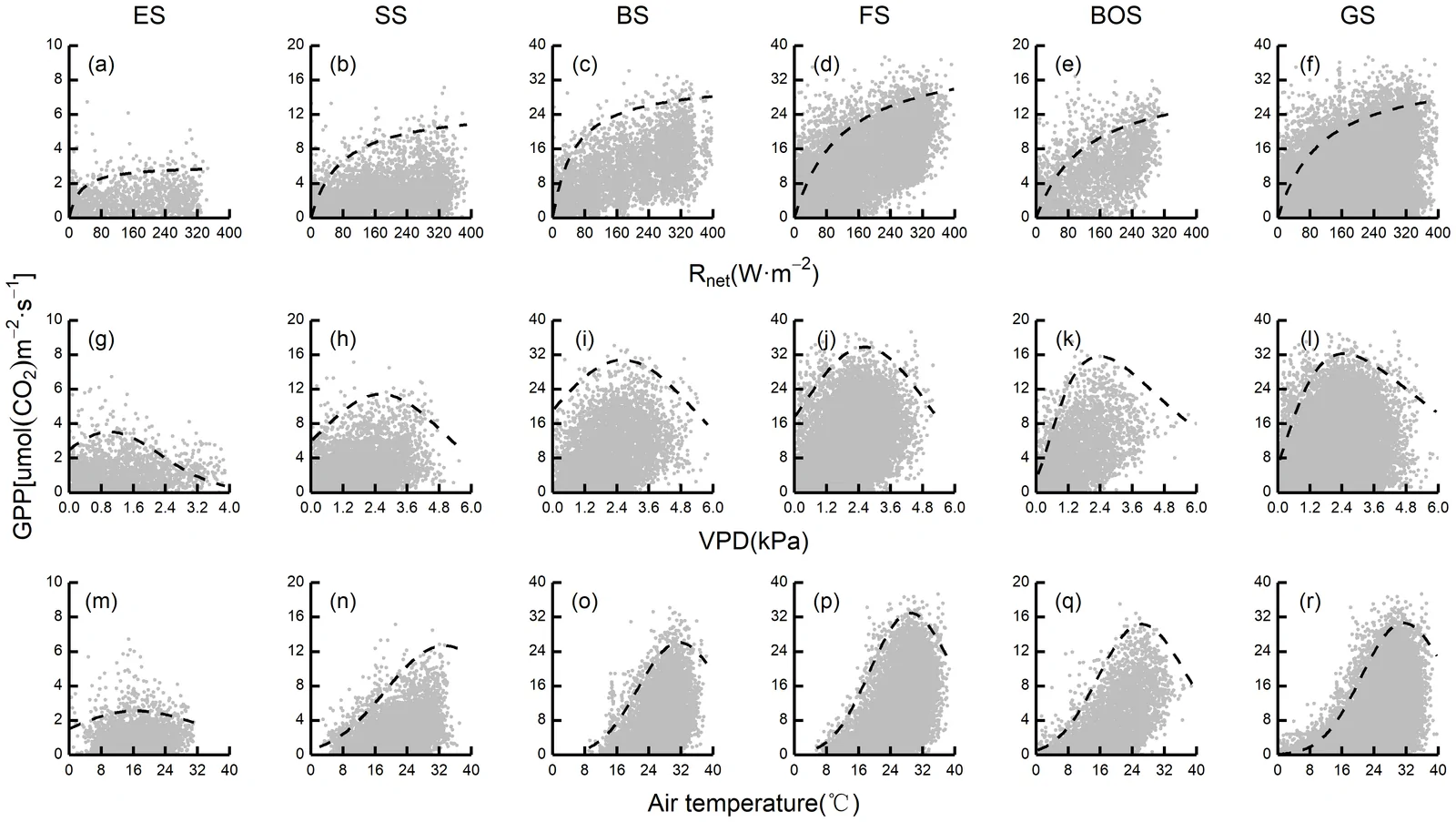
Responses of daytime GPP to R_net_ (**a–e** individual stages; **f** whole season), VPD (**g–k** individual stages; **l** whole season), and T_a_ (**m–q** individual stages; **r** whole season) in the DIPM cotton system. Black dotted lines show 95% confidence intervals.

The response of daytime GPP to R_net_, VPD, and T_a_ displayed distinct seasonal variations, reflecting the transition of physiological sensitivity across different growth stages (**Figure 7**; **Table 1**). The data were selected for daytime (R_net_ > 10 W m^-^²) at each critical growth stage. Across the growing season, GPP exhibited a typical light-saturation pattern, increasing asymptotically with R_net_ (**Figures 7a–f**). The GPP_max_ followed a unimodal seasonal trend, increasing from 3.05 μmol m⁻² s⁻¹ at the ES to a peak of 38.78 μmol m⁻² s⁻¹ during FS. The curvature parameter (*b*), which represents the light-use efficiency sensitivity, varied markedly across growth stages. It was 26.23 W m⁻² at ES, increased to 76.47 W m⁻² at SS, and reached a maximum of 124.04 W m⁻² at BOS. This trend indicates a slower initial rise in GPP per unit R_net_ during peak growth. For the entire growing season (GS), the fitted light-response curve was characterized by an aggregate GPP_max_ of 34.80 μmol m⁻² s⁻¹ and a moderate *b* value of 108.18 W m⁻² (**Table 1**).

**Table 1:** Fitted parameters for the responses of daytime gross primary productivity (GPP, μmol CO_2_ m⁻² s⁻¹) to net radiation (R_net_, W m⁻²), vapor pressure deficit (VPD, kPa), and air temperature (T_a_, °C) at different growth stages of the DIPM cotton system in 2009-2013, 2016-2019, and 2022.

Growth stages	Response of GPP to R_net_		Response of GPP to VPD				Response of GPP to T_a_			
	GPP_max_	b	GPP_max_	K_1_	K_2_	K_3_	GPP_max_	K_1_	K_2_	K_3_
ES	3.05	26.23	3.53	4.20	12.20	1.00	2.56	0.20	107.20	16.56
SS	12.96	76.47	11.43	9.97	104.73	2.62	12.70	0.54	201.00	33.25
BS	32.04	54.23	30.80	8.32	120.18	2.62	26.13	0.73	127.00	31.58
FS	38.78	118.24	33.80	9.40	98.99	2.63	32.95	0.73	126.00	28.96
BOS	16.57	124.04	15.82	0.40	0.99	2.37	15.20	0.73	151.00	26.30
GS	34.80	108.18	32.25	0.32	1.21	2.46	30.65	0.73	136.00	31.38

The response of GPP to VPD and T_a_ followed similar concave functions, identifying specific environmental optima (*K3*) for each stage (**Figures 7g-r**). The optimal VPD (*K3*) increased from 1.00 kPa at ES to 2.62 kPa at SS and BS, and remained high at 2.63 kPa during FS, before decreasing to 2.37 kPa at BOS. This shift suggests an enhanced tolerance to atmospheric water deficit during peak biomass accumulation. For the entire growing season, the cotton system exhibited an integrated optimal VPD of 2.46 kPa, which serves as a threshold for gas exchange under arid conditions, with an aggregated GPP_max_ of 32.25 μmol m⁻² s⁻¹ (**Table 1**).

The optimal T_a_ for photosynthesis also showed a clear seasonal trend. It increased from 16.56 °C at ES to a peak of approximately 33.25 °C at SS. During the mid-season, it remained relatively high at 31.58 °C (BS) and 28.96 °C (FS), before declining to 26.30 °C at BOS. The relative stability of the thermal optimum during BS and FS indicates that the cotton photosynthetic apparatus operated near its physiological peak under the ambient temperature conditions of the oasis. The corresponding GPP_max_ in response to T_a_ peaked at 32.95 μmol m⁻² s⁻¹ during FS. For the entire growing season, the fitted T_a_ response yielded an aggregate optimal temperature of 31.38 °C and a GPP_max_ of 30.65 μmol m⁻² s⁻¹ (**Table 1**), reflecting the long-term adaptation of the DIPM cotton system to the local energy balance and microclimate.

### 3.4 Relative importance of biotic and abiotic drivers for carbon fluxes

**Figure 8:**
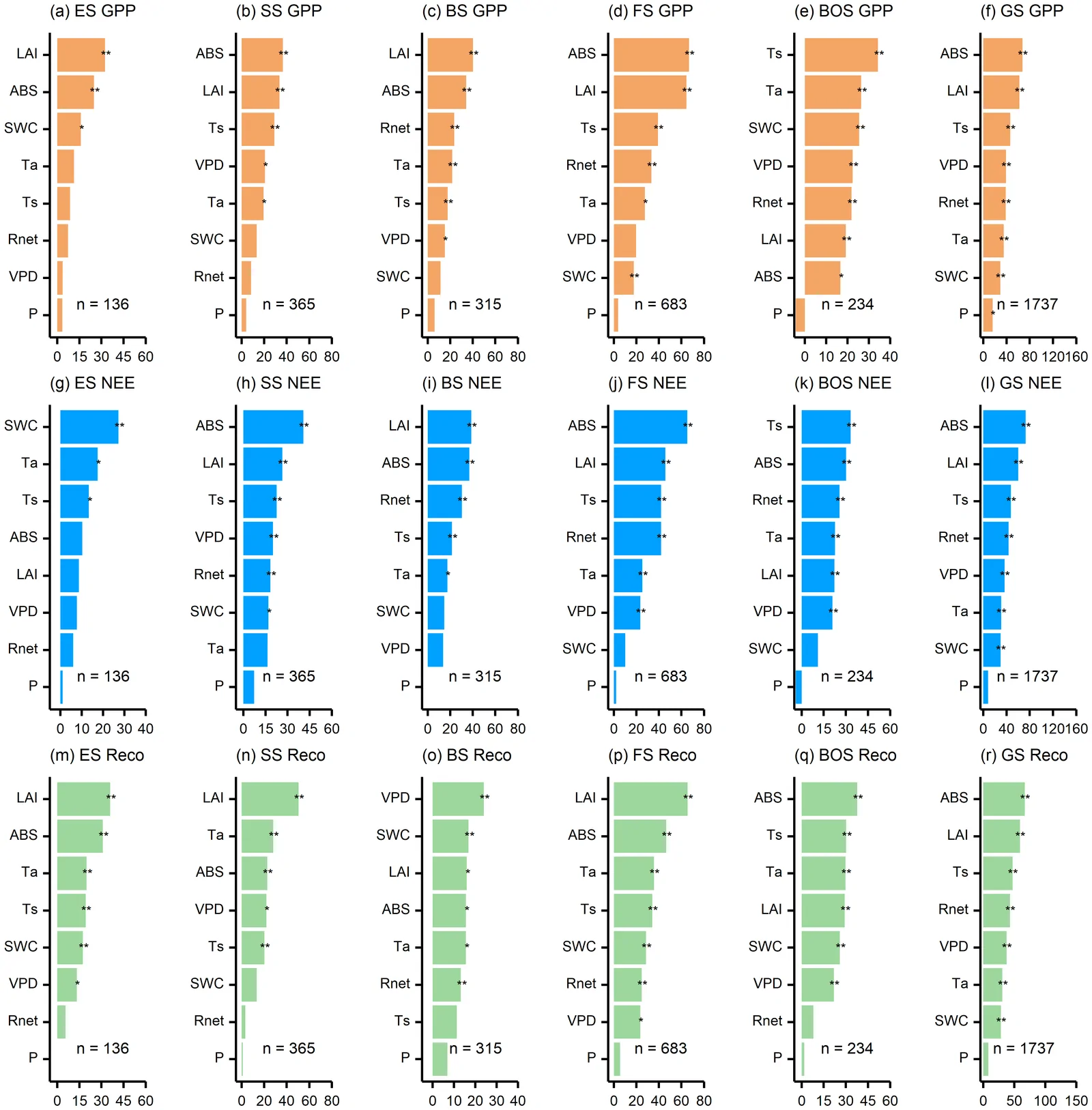
Variable importance in the random forest model for GPP, NEE, and R_eco_ across five growth stages of the DIPM cotton system. Importance values represent the percent increase in mean squared error (% MSE) when each variable is permuted. Asterisks indicate statistical significance (* *p < 0.05*; ** *p < 0.01*). *n* is the number of observations.

Random Forest (RF) models were used to quantify the relative importance of biotic (LAI, ABS) and abiotic drivers for carbon fluxes (GPP, NEE and R_eco_) across the growth stages (**Figure 8**; importance expressed as % increase in MSE). During the early vegetative stages (ES and SS), biotic variables were the primary predictors for all carbon fluxes. LAI and ABS showed substantial contributions, with importance values ranging from 29% to 58%. For GPP, LAI was the dominant predictor at ES (28.95%), while ABS became the leading factor at SS (40.31%), indicating that carbon uptake shifted from canopy structure control to biomass accumulation control as the plants developed. For NEE, SWC was the primary driver at ES (23.26%), reflecting the influence of hydrothermal conditions during the period of minimum canopy cover, whereas ABS emerged as the dominant predictor at SS (35.73%). For R_eco_, LAI was the top predictor at both ES (34.45%) and SS (57.64%), suggesting a close coupling between ecosystem respiration and plant growth during early development.

During the reproductive stages (BS-BOS), the importance of abiotic factors increased, particularly during the late season. For GPP, LAI remained the dominant predictor at BS (39.69%), while VPD, T_s_, and T_a_ became increasingly important at FS and BOS; T_s_ emerged as the leading predictor at BOS (39.70%). For NEE, ABS was the primary driver across all reproductive stages, with importance values of 38.67% (BS), 74.79% (FS), and 32.62% (BOS), indicating that biomass accumulation continued to exert strong control over net carbon uptake during reproductive development. For R_eco_, VPD became the leading predictor at BS (28.09%), while LAI regained dominance at FS (63.67%), and ABS became the top predictor at BOS (39.68%).

The transition from biotic to abiotic control was most evident for GPP: LAI importance decreased from 28.95% at ES to 19.68% at BOS, while T_s_ importance increased from 10.85% at ES to 39.70% at BOS, representing a shift of 28.85 percentage points. Similarly, VPD importance for GPP rose from 3.76% at ES to 23.38% at BOS. Across the full growing season, biotic variables remained the highest-ranked predictors (ABS: 66.38%-73.59%; LAI: 58.97%-63.77%, *p < 0.01*), but T_s_ (33.35%-48.28%) and VPD (36.90%-37.05%) also showed substantial importance (**Figure 8**).

### 3.5 Annual carbon budget and its interannual trends

**Figure 9:**
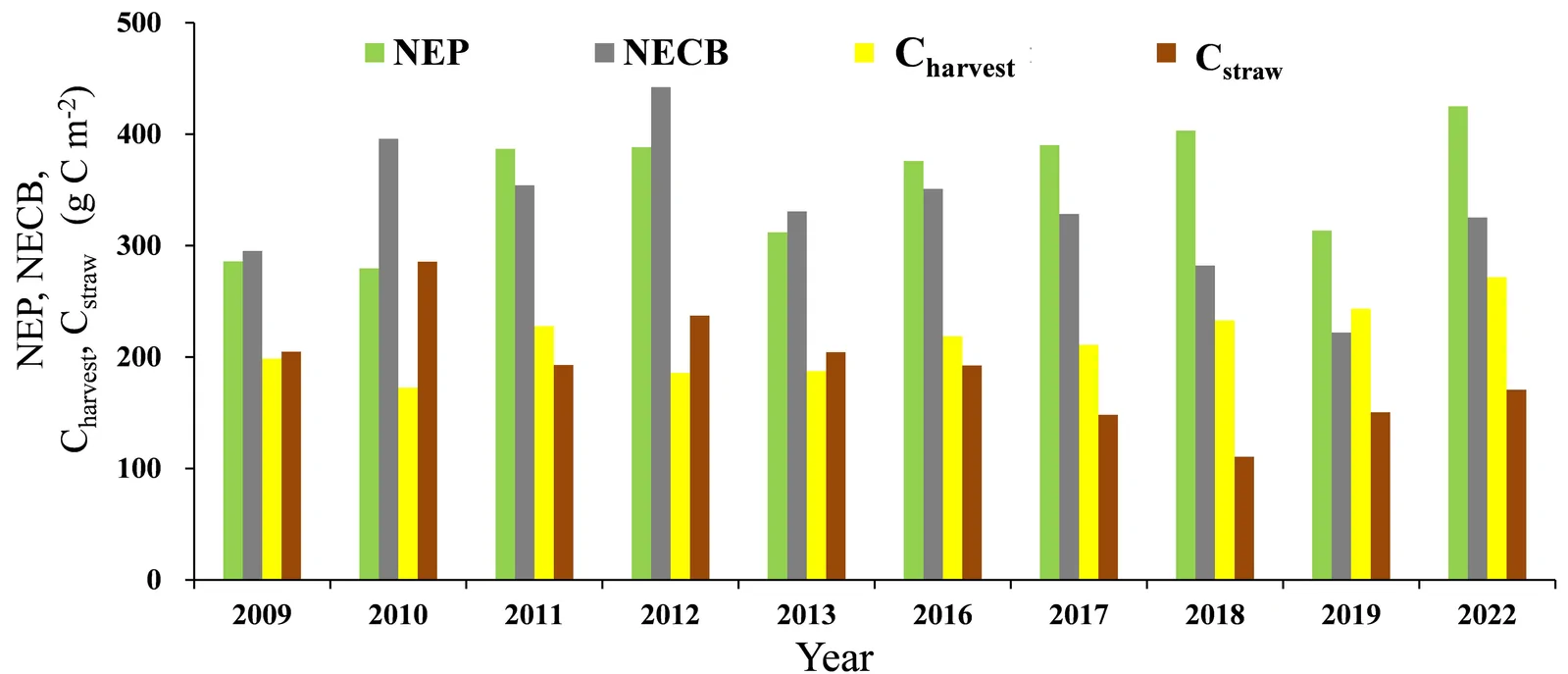
Annual net ecosystem carbon budget (NECB) of the DIPM cotton system. Bars represent net ecosystem production (NEP), carbon input from straw return (C_straw_), and carbon output from seed-cotton harvest (C_harvest_). Units are g C m⁻².

In contrast to the growing-season flux totals (Section 3.2.3), the carbon budget components reported here are annual totals calculated following the NECB framework (Section 2.5). The carbon budget of the DIPM cotton system was estimated by integrating the annual NEP (derived from EC-based NEE as NEP=−∑NEE) with lateral carbon fluxes from seed-cotton harvest export and straw return (**Figure 9**; annual GPP and R_eco_ were provided in **Supplementary Table 3**). Across ten years, annual NEP ranged from 279.61 to 424.85 g C m⁻², indicating consistent net ecosystem carbon gain on an annual basis. Carbon removed through seed-cotton harvest (C_harvest_) varied from 172.53 to 271.47 g C m⁻² (averaging 214.89 ± 28.59 g C m⁻²) while carbon returned to the field as straw (C_straw_) ranged from 110.46 to 285.46 g C m⁻² (mean of 189.64 ± 46.69 g C m⁻²). The resulting annual NECB ranged from 221.88 to 442.18 g C m⁻², with a mean of 332.64 ± 57.52 g C m⁻², confirming that the DIPM system functioned as a net carbon sink.

Linear regression analysis showed distinct interannual trends for each budget component. Annual NEP and C_harvest_ increased at rates of 9.94 g C m⁻² yr⁻¹ (*R*^2^ = 0.33) and 7.78 g C m⁻² yr⁻¹ (*R*^2^ = 0.61), respectively. In contrast, C_straw_ decreased by 11.78 g C m⁻² yr⁻¹ (*R*^2^ = 0.52), leading to a decline in NECB of 9.87 g C m⁻² yr⁻¹ (*R*^2^ = 0.24) over the ten-year period. These trends indicate that the net carbon sink strength of the DIPM system weakened over time, primarily due to reduced residue return to the soil.

## 4 Discussion

### 4.1 Comparison with other cotton management practices

Compared with cotton systems managed without drip irrigation under plastic mulch (non-DIPM), the DIPM cotton system in northern Xinjiang exhibited a stronger growing-season net CO_2_ sink (**Table 2**). Growing-season NEE averaged -383.61 ± 38.51 g C m⁻², exceeding most values reported for non-DIPM cotton systems, including rainfed systems in Texas (-260 to -49 g C m⁻²; Menefee et al., 2020) and India (-362.76 g C m⁻²; Chakraborty et al., 2022), as well as sprinkler-irrigated systems in Shanxi (-226 g C m⁻²; Wang et al., 2013) and Arkansas (-323 g C m⁻²; Fong et al., 2020).

**Table 2:** Comparison of growing-season (or annual) carbon fluxes (NEE, GPP) and carbon budget components (NECB and C_harvest_) between the DIPM cotton system in northern Xinjiang (this study) and other cotton fields reported in the literature.

Site	Climate	Period (month/year)	Irrigation method/amount (mm)	Crop growing days	NEE (g C m^-2^)	GPP (g C m^-2^)	NECB (g C m^-2^)	C_harvest_ (g C m^-2^)	Reference
North Xinjiang, China	Temperate continent	Apr-Oct (2009-2013,2016-2019,2022)	DIPM (427.07 ± 134)	171	-383.61 ± 38.51^a^	861.66 ± 68.02^a^	332.64 ± 57.52^b^	214.89 ± 28.59	This study
North Xinjiang, China	Temperate continent	Apr-Oct (2009-2013)	DIPM (469.88 ± 108)	166	-478.6 ± 41.4^a^	816.2 ± 55.0^a^	NA	NA	Bai et al., 2015
South Xinjiang, China	Arid continent	Annual (2012-2016)	DIPM (565)	160	-101 ± 10 ^b^	1441 ± 20 ^b^	67 ± 37 ^b^	168 ± 27	Ming et al., 2021
Shanxi, China	Temperate monsoon	Apr-Oct (2008)	SI (150.5)	177	-226^a^	NA	NA	NA	Wang et al., 2013,
Shanxi, China	Temperate monsoon	Annual (2008-2010)	SI (188-144)	NA	-36.3 ± 31~8-4.9 ± 34^b^	NA	152.7 ± 22.79~97.4 ± 26.8^b^	195.9 ± 27.7~189.6 ± 26.6	Liu et al., 2019
Michigan, USA	Humid sub-tropical	Apr-Sep (2017)	FI (60)	141	-241.53^a^	NA	NA	49.90*	Anapalli et al., 2019
Arkansas, USA	Humid sub-tropical	May-Oct (2016, 2017)	SI (190-228)	140-171	-323^a^	1370^a^	NA	69.34*	Fong et al., 2020
Texas, USA	Humid sub-tropical	Apr–Sep (2017)	RF	153	-260^a^	947^a^	NA	106*	Menefee et al., 2020
		Apr–Sep (2018)		159	-49^a^	883^a^	NA	54.1*	
Nagpur, India	Tropical dry sub-humid	Jun-Jan (2019-2020,2020-2021)	RF	200-216	-362.76^a^	1027.025^a^	NA	62.63	Chakraborty et al., 2022

Data are mean ± SD or range as reported. This study: *n* = 10 years. NECB: net ecosystem carbon budget; C_harvest_: harvest carbon export. DIPM: drip-irrigated and plastic-mulched; SI: sprinkler irrigation; FI: furrow irrigation; RF: rainfed. NA: not available or not reported.

^a^ Growing season values; ^b^ annual values.

^*^ Lint cotton yield, all other yield values refer to seed-cotton yield.

Within the same WLWS_AES in northern Xinjiang, carbon flux estimates varied between studies. Bai et al. (2015) reported a growing-season NEE of -478.6 ± 41.4 g C m⁻² and GPP of 816.2 ± 55.0 g C m⁻² for 2009-2013, yielding a NEE/GPP ratio of -0.59. In comparison, the present study, covering partially overlapping years, showed higher growing-season GPP (861.66 ± 68.02 g C m⁻²) but less negative growing-season NEE (-383.61 ± 38.51 g C m⁻²) and a growing-season NEE/GPP ratio of -0.45. These discrepancies likely reflect differences in flux data processing, such as gap-filling algorithms and NEE partitioning methods, which are known sources of uncertainty in carbon flux estimates (Reichstein et al., 2005; Wutzler et al., 2018).

A regional comparison between northern and southern Xinjiang reveals contrasting carbon sink strengths. Using annual carbon fluxes from both sites to ensure consistency, the southern cotton field (Ming et al., 2021), warmer and more arid, had much higher annual GPP (1441 ± 20 g C m⁻²) but much lower annual NEP (101 ± 10 g C m⁻²) than the northern site in this study (annual GPP: 965.68 ± 84.45 g C m⁻²; annual NEP: 355.95 ± 50.02 g C m⁻²; **Supplementary Table 3**), reflecting a higher annual R_eco_/GPP ratio (0.93 vs. 0.63).Ming et al. (2023) further partitioned respiration in the same southern field and found that heterotrophic respiration accounted for 52% of R_eco_ and 68% of soil respiration, suggesting that warmer conditions stimulated microbial decomposition. As a result, the southern cotton field was only a weak carbon sink after harvest removal (NECB = 67 ± 37 g C m⁻²), whereas the northern cotton field maintained a strongly positive NECB (332.64 ± 57.52 g C m⁻²), supported by substantial straw return.

The annual NEE/GPP ratio in this study (-0.37) was comparable to the sprinkler-irrigated system in Arkansas (-0.24) and higher than the rainfed systems in Texas (-0.06 to -0.27) and India (-0.35). The stronger net uptake under DIPM can be attributed to the synergistic effects of plastic mulch and drip irrigation. Field experiments have shown that DIPM increased cotton biomass and lint yield by 61.49% and 12.83%, respectively (Zong et al., 2020). Li et al. (2023) and Wang et al. (2020, 2021a) demonstrated that irrigation regimes modify soil hydrothermal conditions, root proliferation, and root-shoot carbon allocation, thereby influencing final yield. Li et al. (2011) reported that transition from flood irrigation to DIPM reduced soil CO_2_ emissions by approximately 100 g C m⁻² yr⁻¹ in this region, due to reduced gas diffusion and increased soil moisture under mulch. The response of carbon pools to DIPM management, however, is not uniform across fractions: optimizing irrigation and nitrogen fertilization may increase soil organic carbon while depleting soil inorganic carbon (Xiao et al., 2024). Plastic mulch reduces evaporation and moderates soil temperature, while drip irrigation supplies water and nutrients more steadily to the root zone (Zhang et al., 2023). These practices improve soil microclimate, sustain canopy development, and prolong the period of high photosynthetic activity during mid-season, contributing to the enhanced carbon sink strength observed in this system.

Finally, the positive NECB of the cotton field in northern Xinjiang, coupled with substantial harvest carbon export (C_harvest_ = 214.89 ± 28.59 g C m⁻²), demonstrates that enhanced CO_2_ uptake was effectively converted into economic yield while maintaining a positive ecosystem carbon balance. In contrast, the rainfed cotton systems in Texas and India had much lower C_harvest_ values (54–106 g C m⁻² as lint and 62.63 g C m⁻² as seed-cotton), highlighting less efficient carbon conversion under water-limited conditions.

### 4.2 Environmental and management controls on interannual carbon budget dynamics

**Figure 10:**
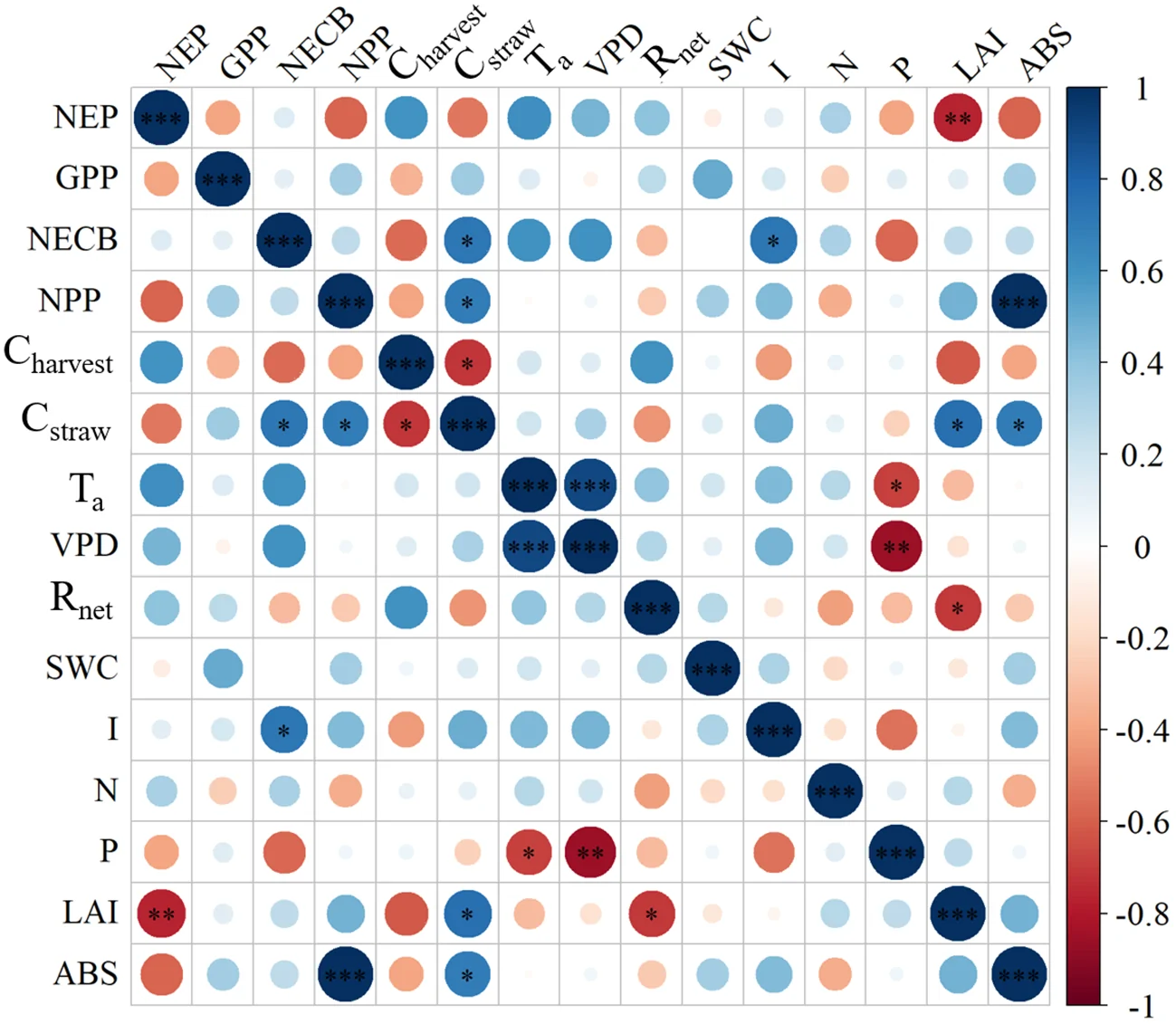
Correlation matrix among annual NEP, GPP, NECB, NPP, harvest carbon (C_harvest_), straw carbon (C_straw_), T_a_, VPD, R_net_, SWC, I, nitrogen fertilization rate (N), precipitation (P), LAI, and ABS for the DIPM cotton system. Blue indicates positive correlation, red indicates negative correlation. Color intensity represents correlation strength. Asterisks indicate significance levels (* *p < 0.05*; ** *p < 0.01*; *** *p < 0.001*).

The weakening of NECB over the ten-year period was primarily driven by the decline in straw return carbon (C_straw_, decreased by 11.78 g C m⁻² over the study period), as indicated by the strong positive correlation between NECB and C_straw_ (*r* = 0.72, *P = 0.02*) (**Figure 10**). The negative correlation between C_straw_ and C_harvest_ (*r* = -0.71, *P = 0.09*) suggests a trade-off: increasing harvest intensity over time reduced the amount of residue available for return. Similar trade-offs between yield improvement and straw return have been documented in other cropping systems (Wang et al., 2015; Liu et al., 2019). This trend likely reflects a shift in management priorities toward maximizing economic yield at the expense of residue retention.

The increase in NEP (9.94 g C m⁻²) showed positive trends with T_a_ (*r* = 0.61, *P = 0.06*), VPD (*r* = 0.47, *P = 0.17*), and R_net_ (*r* = 0.41, *P = 0.24*), though none of these relationships reached statistical significance. In contrast, the correlation between NEP and LAI was much stronger and statistically significant (*r* = –0.77, *P = 0.01*) (**Figure 10**). This contrast indicates that canopy development, rather than direct climatic forcing, was the primary mediator of interannual NEP variability. This finding is consistent with multiple cropland studies across China. In the North China Plain, LAI was found to be the most important driver of CO_2_ fluxes during the wheat season in winter wheat-summer maize rotation systems (Yue et al., 2023). Similarly, in the Guanzhong Plain, LAI was confirmed as the primary control on the seasonal variation of NEE and the dominant direct driver of GPP in summer maize and winter wheat croplands (Peng et al., 2023). Furthermore, LAI has been reported as a key control on water–carbon fluxes and the dominant control on total ecosystem respiration in cropland ecosystems (Liu et al., 2026). Collectively, these cross-site findings support the conclusion that canopy development (LAI), rather than the direct effects of climatic factors, was the dominant driver of carbon sink variability in the DIPM cotton system studied here.

Irrigation amount showed a strong positive correlation with NECB (*r* = 0.73, *P = 0.02*), highlighting the importance of water input for maintaining net carbon gain. Adequate irrigation improved soil water conditions, which promoted fine root growth and biomass accumulation, regulated root-shoot relations, and suppressed excessive vegetative growth (Wang et al., 2020). Meanwhile, DIPM maintained photosynthetic capacity by moderating soil hydrothermal conditions (Zhou et al., 2012). However, irrigation and mulching can also increase soil temperature and moisture, thereby stimulating CO_2_ emissions (Zong et al., 2020; Ming et al., 2023), so the net effect of irrigation on NECB depends on the balance between enhanced carbon uptake and stimulated carbon release (Ming et al., 2023). In contrast, no statistically significant correlations were found between SWC and carbon budget components. Irrigation amount integrates the cumulative water supply over the growing season, whereas SWC measured at 20 and 40 cm depths represents only a transient soil water status that is rapidly depleted by crop uptake and evapotranspiration following each irrigation event (Zhou et al., 2012). Thus, irrigation amount is a more integrative measure of water availability than SWC, indicating that carbon gain in DIPM systems is more responsive to management-driven water input than to static soil moisture conditions. Precipitation showed a negative correlation with NECB (*r* = -0.57, *P = 0.08*), further supporting the decoupling of the DIPM system from rainfall through irrigation management (Li et al., 2011). However, higher irrigation does not always benefit carbon sequestration. Excessive irrigation can promote excessive vegetative growth, reduce boll opening rate and yield (Wang et al., 2020), and increase CO_2_ emissions and nitrate leaching, potentially offsetting carbon gains (Xiao et al., 2024). Optimizing irrigation to balance carbon uptake, yield, and environmental costs is therefore critical for sustainable DIPM management.

### 4.3 Carbon allocation shift under DIPM and its implications for the carbon budget

**Figure 11:**
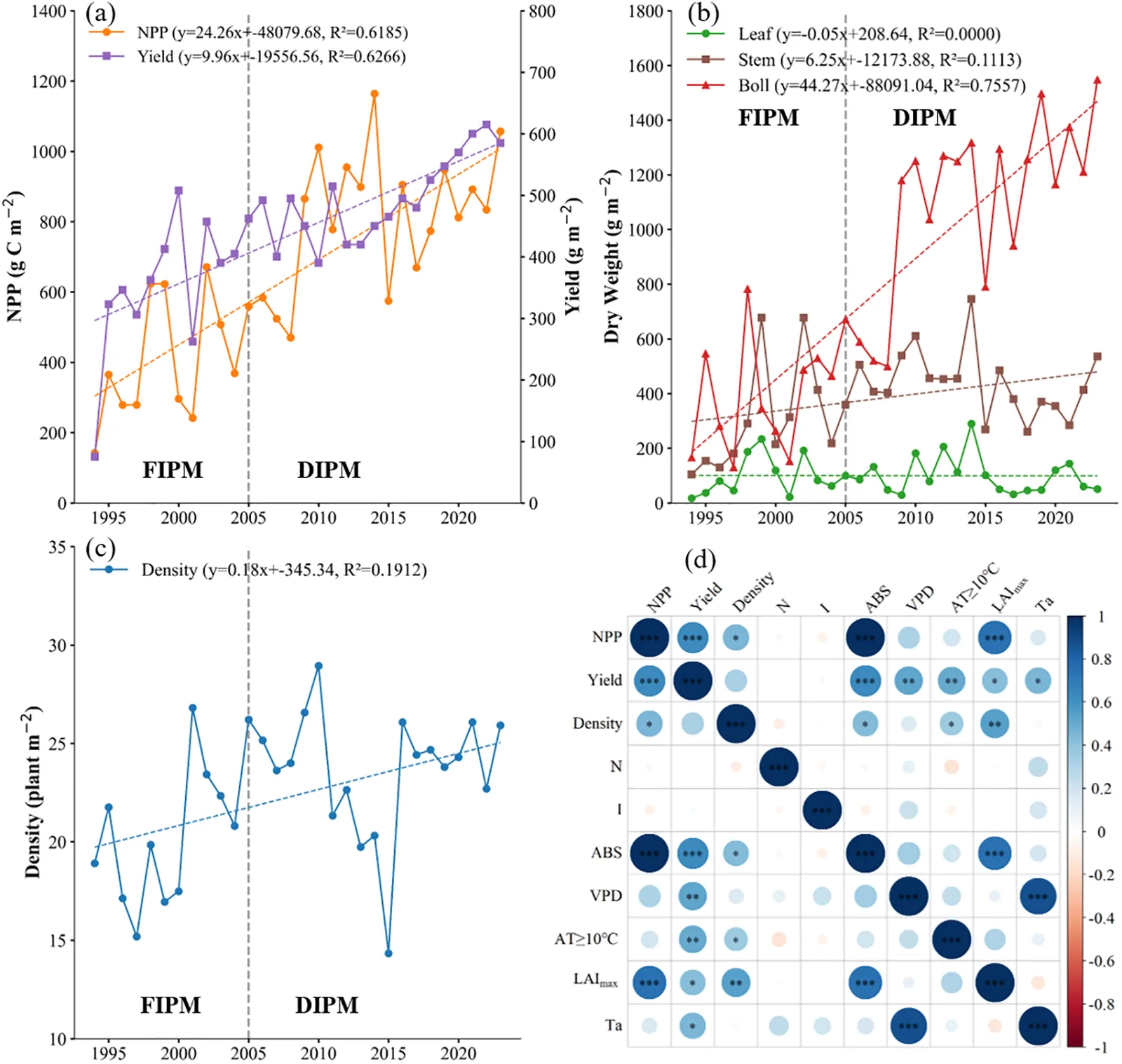
Long-term dynamics (1994-2023) of cotton NPP, yield, biomass allocation, and planting density at WLWS_AES, and the contributions of climatic and management factors to NPP variability. **(a)** Dynamics of NPP and seed-cotton yield. The vertical dashed line separates the furrow-irrigated and plastic-mulched (FIPM) period (1994-2005) from the drip-irrigated and plastic-mulched (DIPM) period (2006-2023). **(b)** Aboveground dry biomass allocation among bolls, stems, and leaves. **(c)** Cotton planting density. **(d)** Relative importance of climatic factors (T_a_, AT≥10°C, VPD) and management factors (I, N, planting density) for the interannual variability of NPP. Asterisks indicate significance levels (* *p < 0.05*; ** *p < 0.01*; *** *p < 0.001*).

The long-term records at WLWS_AES (1994-2023) reveal a fundamental shift in carbon allocation following the transition from FIPM to DIPM (**Figure 11**). The long-term biomass records were also collected from WLWS_AES. NPP was estimated using the same method described in Section 2.5 (based on ABS and a root-to-shoot ratio of 0.11), ensuring consistency between the long-term trends and the EC-based carbon budget analysis. Mean NPP increased by 104% (from 401.56 ± 180.09 to 817.61 ± 185.42 g C m⁻²) and seed-cotton yield by 38% (from 359.20 ± 109.04 to 495.25 ± 66.49 g m⁻²) under DIPM (**Table 3**; **Figure 11a**). This productivity enhancement is corroborated by long-term evidence from Xinjiang’s saline-alkali cotton fields, where continuous drip fertigation under plastic mulch over 12–27 years increased cotton yield from 5272 to 6102 kg ha⁻¹ (Luo et al., 2026). Planting density increased by 15% (from 20.57 ± 3.54 to 23.59 ± 3.15 plants m⁻²), while irrigation amount decreased by 22% (from 544.67 ± 217.24 to 425.82 ± 118.27 mm) under DIPM (**Table 3**; **Figures 11a, c, d)**. Consistent with this, Wu et al. (2024) demonstrated that deficit irrigation combined with high planting density (22.5 plants m⁻²) achieved 20% water savings without sacrificing seed cotton yield in Xinjiang cotton fields.

**Table 3:** Comparison of key indicators between FIPM (1994-2005) and DIPM (2006-2023) at the WLWS_AES site.

Indicator	FIPM (1994-2005)	DIPM (2006-2023).
NPP (g C m⁻²)	401.56 ± 180.09	817.61 ± 185.42
C_harvest_ (g C m⁻²)	150.45 ± 28.26	196.12 ± 26.33
Seed-cotton yield (g m⁻²)	359.20 ± 109.04	495.25 ± 66.49
ABS (g m⁻²)	834.43 ± 341.10	1651.73 ± 374.58
Boll proportion (%)	0.48 ± 0.15	0.66 ± 0.09
Stem proportion (%)	0.37 ± 0.13	0.27 ± 0.08
Leaf proportion (%)	0.11 ± 0.05	0.06 ± 0.03
Planting density (plants m⁻²)	20.57 ± 3.54	23.59 ± 3.15
Irrigation amount (mm)	544.67 ± 217.24	425.82 ± 118.27
Nitrogen fertilization (g N m⁻²)	18.27 ± 1.18	16.88 ± 11.29

ABS allocation shifted markedly toward reproductive organs: the boll proportion increased from 0.48 ± 0.15 to 0.66 ± 0.09, while the stem and leaf proportions decreased from 0.37 ± 0.13 to 0.27 ± 0.08 and from 0.11 ± 0.05 to 0.06 ± 0.03, respectively (**Table 3**; **Figure 11b**). This reallocation pattern is consistent with the optimized root-shoot relationship under drip irrigation reported by Wang et al. (2020; Wang et al., 2021a), who found that mulched drip irrigation promoted assimilate partitioning to reproductive organs through regulation of root-shoot dynamics. The same authors further demonstrated that improved fine root plasticity enhances root absorptive capacity, thereby supporting higher yield with reduced water consumption (Wang et al., 2021a). Hu et al. (2026) recently confirmed this pattern in saline-alkali cotton fields in Xinjiang, showing that moderate leaching under drip irrigation effectively alleviated salinity stress and promoted biomass accumulation and reproductive allocation during critical growth stages. Consistent with these findings, Wu et al. (2024) demonstrated that deficit irrigation combined with high planting density achieved 20% water savings while maintaining yield stability through enhanced dynamic consistency of root distribution and soil water-nitrogen consumption. Collectively, these studies confirm that DIPM fundamentally alters carbon partitioning by favoring reproductive over vegetative growth.

This shift has dual implications for the carbon budget. On the positive side, the increased boll proportion contributed to a 30% increase in harvest carbon export (C_harvest_, from 150.45 ± 28.26 to 196.12 ± 26.33 g C m⁻²; **Table 3**; **Figure 11d**) and supported a strongly positive NECB (332.64 ± 57.52 g C m⁻²). On the negative side, the reduced stem and leaf proportions reduced the vegetative biomass available for straw return, reflected in the decline in C_straw_ under DIPM and the positive correlation between C_straw_ and LAI (*r* = 0.75, *P = 0.01*; Section 3.5). A national-scale meta-analysis further revealed that while straw return significantly increases soil organic carbon (by approximately 14%), this effect may diminish or even reverse after approximately 10 years of continuous practice (Wang et al., 2021b). This suggested that the sustained decline in residue return under DIPM could threaten long-term soil carbon sequestration, as the system might lose the capacity to maintain or increase SOC stocks over time. Thus, DIPM achieved higher productivity with reduced irrigation and nitrogen imputed but at the cost of reduced residue return, a trade-off consistent with findings from other cropping systems where improvements in harvest index often come at the expense of residue retention (Wang et al., 2015; Liu et al., 2019). To sustain long-term soil carbon sequestration under DIPM, additional measures that enhance residue retention, such as optimizing harvest intensity, incorporating cover crops, or adjusting plant density, might be necessary.

### 4.4 Uncertainty analysis for NECB

The uncertainty of NECB arises from multiple sources. Here we consider three main sources: (1) uncertainty in NEE gap-filling, which propagates to NEP; (2) uncertainty in the root-to-shoot ratio (*R_r_s_*) used for NPP estimation; and (3) uncertainty in the straw return fraction (*f_R_*) used for C_straw_ calculation. Each source was quantified separately and then combined to provide an overall uncertainty estimate for NECB.

#### 4.4.1 Uncertainty from NEE gap-filling

Data gap filling is a major source of uncertainty, particularly when flux data gaps exceed 7 days. Following Wutzler et al. (2018), we constructed four imputation scenarios with varying thresholds for R_g_, T_a_, and RH (**Table 4**). Compared to the baseline scenario (annual NEE = -355.95 g C m⁻²), annual NEE ranged from -333.76 ± 45.84 g C m⁻² (strict conditions) to -363.20 ± 46.84 g C m⁻² (very lenient conditions), with deviations of +22.19 g C m⁻² (+6.2%) and -7.25 g C m⁻² (-2.0%), respectively. All scenarios confirmed the ecosystem as a net carbon sinks.

**Table 4:** Annual NEE results of the DIPM cotton system under different environmental factor threshold settings.

Scenario	R_g_	T_a_	RH	NEE (g C m^-^²)	Description
I	± 25	± 1	± 5	-333.76 ± 45.84	Strict conditions
II	± 50	± 2	± 10	-355.95 ± 50.02	Moderate conditions (This study)
III	± 75	± 3	± 15	-361.41 ± 45.26	Loose conditions
IV	± 100	± 5	± 20	-363.20 ± 46.84	Super-loose conditions

#### 4.4.2 Uncertainty from carbon budget parameters (*R_r_s_* and *f_R_*)

In addition to flux gap-filling uncertainties, the NECB calculation involves several assumptions that may introduce additional uncertainty. Two key parameters are the root-to-shoot ratio (*R_r_s_* = 0.11) used to estimate NPP from ABS, and the straw return fraction (*f_R_* = 0.85) used to calculate C_straw_. Both were treated as constants due to the lack of interannual measurements. To quantify the sensitivity of NECB to these parameters, a scenario-based analysis was conducted by varying *R_r_s_* at ±10%, ± 20%, and ±30% of its baseline value, and *f_R_* at 80%, 85% and 90%. These ranges were chosen based on the observed variability in *R_r_s_* across different yield levels and moisture conditions in Xinjiang cotton fields (Zhang et al., 2015; Gao et al., 2020), and the reported range of straw return fractions in the region.

Varying *R_r_s_* altered the multi-year mean NECB by ±1.5% to ±4.5%, while varying *f_R_* between 80% and 90% changed NECB by -3.4% to +3.4%. Under the most conservative combination (*R_r_s_* increased by 30% and *f_R_* = 80%), the multi-year mean NECB decreased from 332.65 to 308.40 g C m⁻², a reduction of 7.3%, but remained positive (**Table 5**).

**Table 5:** Sensitivity of multi-year mean NECB to uncertainties in root-to-shoot ratio (*R_r_s_*) and straw return fraction (*f_R_*).

Parameter	Scenario	Parameter value	Change in NECB (%)
Root-to-shoot ratio (*R_r_s_*)	Baseline	0.11	—
	+10%	0.121	+1.5
	−10%	0.099	−1.5
	+20%	0.132	+3.0
	−20%	0.088	−3.0
	+30%	0.143	+4.5
	−30%	0.077	−4.5
Straw return fraction (*f_R_*)	Baseline	0.85	—
	−5%	0.80	−3.4
	+5%	0.90	+3.4
Most conservative combination	*R_r_s_* increased by 30% and *f_R_* = 80%	—	−7.3

#### 4.4.3 Combined uncertainty for NECB

To provide an overall uncertainty estimate for NECB, the three main sources identified above were combined using first-order error propagation (see Appendix S1 for detailed calculations). The combined relative uncertainty for the multi-year mean NECB was approximately ±16%, corresponding to a standard deviation of ±53.4 g C m⁻² for the mean NECB of 332.64 g C m⁻². Assuming a normal distribution, the 95% confidence interval for the mean NECB is approximately 225.8-439.5 g C m⁻² (332.64 ± 106.8). The positive NECB estimate was robust to uncertainties from both flux data processing and parameter assumptions.

It is important to distinguish between the absolute contribution of straw return to NECB and its contribution to uncertainty. Straw return accounted for approximately 57% of the mean NECB, confirming its critical role in sustaining the positive carbon balance, whereas the main source of uncertainty was NEE gap-filling (87.7%) (**Table 6**), with straw return fraction contributing only about 4.4% (**Table 6**). Thus, improving flux measurement accuracy would most effectively reduce uncertainty, while maintaining straw return remains essential for sustaining the carbon sink. In addition, the NECB estimate does not include potential inorganic carbon inputs via drip irrigation water, a pathway that could influence soil inorganic carbon dynamics but requires water chemistry data not available in this study (Bughio et al., 2016; Kim et al., 2020). Future research combining irrigation water monitoring with soil inorganic carbon measurements would help close this gap.

**Table 6:** Summary of uncertainty sources for NECB.

Uncertainty source	Baseline value	Uncertainty range	Contribution to NECB uncertainty
Annual NEP (from NEE gap-filling)	355.95g C m⁻²	CV = 14% (SD = 50.02 g C m⁻²)	87.7%
Root-to-shoot ratio (*R_r_s_*)	0.11	± 30% (0.077-0.143)	7.7%
Straw return fraction (*f_R_*)	0.85	80%-90%	4. 4%
Combined (NECB)	332.64 g C m⁻²	± 16% (SD = 53.4 g C m⁻²)	100%

## 5 Conclusion

Over ten growing seasons, the DIPM cotton system in arid northern Xinjiang consistently functioned as a growing-season CO_2_ sink (NEE: -383.61 ± 38.51 g C m⁻²). Our findings confirm the hypothesized shift in carbon flux drivers from biotic to abiotic factors across developmental stages, and support the critical role of straw return in sustaining the positive NECB of this system. Net carbon uptake was largely concentrated during the reproductive period, with the flowering stage contributing most to the seasonal carbon sink. During this period, biotic factors, particularly canopy development (LAI) and biomass accumulation (ABS), dominated carbon exchange, whereas abiotic factors (VPD, temperature, and soil water) became increasingly important during late reproduction. Carbon-budget accounting revealed a trade-off: DIPM increased harvest carbon export (C_harvest_) but reduced residue return (C_straw_). Despite this reduction, straw return remained the key management practice sustaining a strongly positive NECB (mean 332.64 ± 57.52 g C m⁻²). Uncertainty analysis confirmed the robustness of this positive NECB, with a 95% confidence interval of 225.8-439.4 g C m⁻². To sustain the carbon sink, we recommend maintaining a straw return fraction of at least 85% and alleviating VPD stress during flowering through optimized irrigation management. Biotic factors, including canopy development and carbon allocation, dominate carbon fluxes and budget in this arid DIPM cotton system, with straw return serving as the essential management lever.

## Data Availability

The original contributions presented in the study are included in the article/**Supplementary Material**. Further inquiries can be directed to the corresponding author.

## References

[B1] Anapalli S. S. Fisher D. K. Reddy K. N. Krutz J. L. Pinnamaneni S. R. Sui R. (2019). Quantifying water and CO_2_ fluxes and water use efficiencies across irrigated C_3_ and C_4_ crops in a humid climate. Sci. Total Environ. 663, 338–350. doi: 10.1016/j.scitotenv.2018.12.471 30716624

[B2] Bai J. Wang J. Chen X. Luo G. Shi H. Li L. . (2015). Seasonal and inter-annual variations in carbon fluxes and evapotranspiration over cotton field under drip irrigation with plastic mulch in an arid region of Northwest China. J. Arid. Land 7, 272–284. doi: 10.1007/s40333-014-0012-x 30311153

[B3] Baldocchi D. Falge E. Wilson K. (2001). A spectral analysis of biosphere–atmosphere trace gas flux densities and meteorological variables across hour to multi-year time scales. Agric. For. Meteorol. 107, 1–27. doi: 10.1016/S0168-1923(00)00228-8

[B4] Béziat P. Ceschia E. Dedieu G. (2009). Carbon balance of a three crop succession over two cropland sites in South West France. Agric. For. Meteorol. 149, 1628–1645. doi: 10.1016/j.agrformet.2009.05.004 38826717

[B5] Bughio M. A. Wang P. Meng F. Qing C. Kuzyakov Y. Wang X. . (2016). Neoformation of pedogenic carbonates by irrigation and fertilization and their contribution to carbon sequestration in soil. Geoderma 262, 12–19. doi: 10.1016/j.geoderma.2015.08.003 38826717

[B6] Chakraborty A. Venugopalan M. V. Mani J. K. Bagadkar A. J. Manikandan A. (2022). Rainfed cotton crop in central India is a strong net CO_2_ sink: An eddy covariance-based analysis of ecosystem fluxes. Field Crops Res. 286, 108595. doi: 10.1016/j.fcr.2022.108595 38826717

[B7] Chen X. Xi K. Yang Z. Lu J. Zhang Q. Wang B. . (2023). Long-term increases in continuous cotton yield and soil fertility following the application of cotton straw and organic manure. Agronomy 13, 2133. doi: 10.3390/agronomy13082133 30654563

[B8] Falge E. Baldocchi D. Olson R. Anthoni P. Aubinet M. Bernhofer C. . (2001). Gap filling strategies for defensible annual sums of net ecosystem exchange. Agric. For. Meteorol. 107, 43–69. doi: 10.1016/S0168-1923(00)00225-2

[B9] Fong B. N. Reba M. L. Teague T. G. Runkle B. R. Suvočarev K. (2020). Eddy covariance measurements of carbon dioxide and water fluxes in US mid-south cotton production. Agric. Ecosyst. Environ. 292, 106813. doi: 10.1016/j.agee.2019.106813 38826717

[B10] Friedlingstein P. O'Sullivan M. Jones M. W. Andrew R. M. Hauck J. Landschützer P. . (2025). Global carbon budget 2024. Earth Syst. Sci. Data 17, 965–1039. doi: 10.5194/essd-17-965-2025

[B11] Gao H. Y. Li J. H. Wang Y. Y. Xia J. Shi X. J. Hao X. Z. . (2020). Response of photosynthetic characteristics and dry matter accumulation to drought of two cotton varieties with different drought resistance. Xinjiang Agric. Sci. 57, 233–244. (in Chinese with English abstract).

[B12] Grant R. F. Arkebauer T. J. Dobermann A. Hubbard K. G. Schimelfenig T. T. Suyker A. E. . (2007). Net biome productivity of irrigated and rainfed maize–soybean rotations: Modeling vs. measurements. Agron. J. 99, 1404–1423. doi: 10.2134/agronj2006.0308

[B13] Hollinger S. E. Bernacchi C. J. Meyers T. P. (2005). Carbon budget of mature no-till ecosystem in North Central Region of the United States. Agric. For. Meteorol. 130, 59–69. doi: 10.1016/j.agrformet.2005.01.005 38826717

[B14] Hu Q. Y. Cao H. X. He Z. J. Shi H. L. Qi C. Sun S. K. (2026). Cotton yield stability and fiber quality as affected by leaching amounts and drip irrigation types in saline-alkali soils of Xinjiang, China. Agric. Water Manage. 329, 110355. doi: 10.1016/j.agwat.2026.110355 38826717

[B15] Kim J. H. Jobbágy E. G. Richter D. D. Trumbore S. E. Jackson R. B. (2020). Agricultural acceleration of soil carbonate weathering. Global Change Biol. 26, 5988–6002. doi: 10.1111/gcb.15207 32511819

[B16] Lasslop G. Reichstein M. Papale D. Richardson A. D. Arneth A. Barr A. . (2010). Separation of net ecosystem exchange into assimilation and respiration using a light response curve approach: critical issues and global evaluation. Global Change Biol. 16, 187–208. doi: 10.1111/j.1365-2486.2009.02041.x 40046247

[B17] Li Y. W. Chai Y. W. Ma J. T. Li R. Cheng H. B. Chang L. . (2022). Straw strip mulching in a semiarid rainfed agroecosystem achieves carbon sequestration and emission reduction from winter wheat fields. Agric. Ecosyst. Environ. 334, 107990. doi: 10.1016/j.agee.2022.107990 38826717

[B18] Li X. Liu L. Yang H. Li Y. (2018). Relationships between carbon fluxes and environmental factors in a drip-irrigated, film-mulched cotton field in arid region. PLoS One 13, e0192467. doi: 10.1371/journal.pone.0192467 29415018 PMC5802936

[B19] Li Z. P. Wan S. M. Chen G. D. Han Y. C. Lei Y. P. Ma Y. Z. . (2023). Effects of irrigation regime on soil hydrothermal microenvironment, cotton biomass, and yield under non-film drip irrigation system in cotton fields in southern Xinjiang, China. Ind. Crops Prod. 198, 116738. doi: 10.1016/j.indcrop.2023.116738 38826717

[B20] Li Z. G. Zhang R. H. Wang X. J. Wang J. P. Zhang C. P. Tian C. Y. (2011). Carbon dioxide fluxes and concentrations in a cotton field in northwestern China: Effects of plastic mulching and drip irrigation. Pedosphere 21, 178–185. doi: 10.1016/S1002-0160(11)60116-1

[B21] Liu X. A. Li Y. Xu X. Y. Peng X. B. Gu X. B. Yu L. Y. . (2026). Environmental controls on water-carbon fluxes in winter wheat cropland under different hydrological and extreme environmental conditions in a dry semi-humid region. Ecol. Indic. 187, 114918. doi: 10.1016/j.ecolind.2026.114918 38826717

[B22] Liu Z. H. Wang B. F. Li Z. Y. Huang F. Y. Zhao C. X. Zhang P. . (2022). Plastic film mulch combined with adding biochar improved soil carbon budget, carbon footprint, and maize yield in a rainfed region. Field Crops Res. 284, 108574. doi: 10.1016/j.fcr.2022.108574 38826717

[B23] Liu C. Y. Yao Z. S. Wang K. Zheng X. H. Li B. G. (2019). Net ecosystem carbon and greenhouse gas budgets in fiber and cereal cropping systems. Sci. Total Environ. 647, 895–904. doi: 10.1016/j.scitotenv.2018.08.048 30096677

[B24] Luo P. C. Chen R. Yang J. J. Javed T. Zhang J. H. Zhang J. Z. . (2026). Long-term drip fertigation under plastic mulch improves root-zone soil multifunctionality and cotton productivity in a saline-alkali cropland through salinity reduction and structural recovery. Ind. Crops Prod. 244, 123209. doi: 10.1016/j.indcrop.2026.123209 38826717

[B25] Menefee D. Rajan N. Cui S. Bagavathiannan M. Schnell R. West J. (2020). Carbon exchange of a dryland cotton field and its relationship with PlanetScope remote sensing data. Agric. For. Meteorol. 294, 108130. doi: 10.1016/j.agrformet.2020.108130 38826717

[B26] Ming G. H. Hu H. C. Tian F. Q. Khan M. Y. A. Zhang Q. (2021). Carbon budget for a plastic-film mulched and drip-irrigated cotton field in an oasis of Northwest China. Agric. For. Meteorol. 306, 108447. doi: 10.1016/j.agrformet.2021.108447 38826717

[B27] Ming G. H. Zhang Q. Gong W. Wang B. Q. Hu H. C. Tian F. Q. (2023). The characteristics of ecosystem respiration and its components of a representative film-mulched and drip-irrigated cotton field in Northwest China. Agric. Ecosyst. Environ. 352, 108506. doi: 10.1016/j.agee.2023.108506 38826717

[B28] Peng X. B. Wang Y. F. Ma J. Liu X. A. Gu X. B. Cai H. J. (2023). Seasonal variation and controlling factors of carbon balance over dry semi-humid cropland in Guanzhong Plain. Eur. J. Agron. 149, 126912. doi: 10.1016/j.eja.2023.126912 38826717

[B29] Qin R. Z. Guan K. Peng B. Zhang F. Zhou W. Tang J. . (2025). A model-data fusion approach for quantifying the carbon budget in cotton agroecosystems across the United States. Agric. For. Meteorol. 363, 110407. doi: 10.1016/j.agrformet.2025.110407 38826717

[B30] Reichstein M. Falge E. Baldocchi D. Papale D. Aubinet M. Berbigier P. . (2005). On the separation of net ecosystem exchange into assimilation and ecosystem respiration: review and improved algorithm. Global Change Biol. 11, 1424–1439. doi: 10.1111/j.1365-2486.2005.001002.x 40046247

[B31] Smith P. Lanigan G. Kutsch W. L. Buchmann N. Eugster W. Aubinet M. . (2010). Measurements necessary for assessing the net ecosystem carbon budget of croplands. Agric. Ecosyst. Environ. 139, 302–315. doi: 10.1016/j.agee.2010.04.004 38826717

[B32] Snyder C. S. Bruulsema T. W. Jensen T. L. Fixen P. E. (2009). Review of greenhouse gas emissions from crop production systems and fertilizer management effects. Agric. Ecosyst. Environ. 133, 247–266. doi: 10.1016/j.agee.2009.04.021 38826717

[B33] Tao F. L. Li Y. B. Chen Y. Yin L. C. Zhang S. (2022). Daily, seasonal and inter-annual variations in CO_2_ fluxes and carbon budget in a winter-wheat and summer-maize rotation system in the North China Plain. Agric. For. Meteorol. 324, 109098. doi: 10.1016/j.agrformet.2022.109098 38826717

[B34] Vickers D. Mahrt L. (1997). Quality control and flux sampling problems for tower and aircraft data. J. Atmos. Oceanic Technol. 14, 512–526. doi: 10.1175/1520-0426(1997)014<0512:QCAFSP>2.0.CO;2

[B35] Wang J. T. Du G. F. Tian J. S. Zhang Y. L. Jiang C. D. Zhang W. F. (2020). Effect of irrigation methods on root growth, root-shoot ratio and yield components of cotton by regulating the growth redundancy of root and shoot. Agric. Water Manage. 234, 106120. doi: 10.1016/j.agwat.2020.106120 38826717

[B36] Wang J. T. Du G. F. Tian J. S. Jiang C. D. Zhang Y. L. Zhang W. F. (2021a). Mulched drip irrigation increases cotton yield and water use efficiency via improving fine root plasticity. Agric. Water Manage. 255, 106992. doi: 10.1016/j.agwat.2021.106992 38826717

[B37] Wang Y. Y. Hu C. S. Dong W. X. Li X. X. Zhang Y. M. Qin S. P. . (2015). Carbon budget of a winter-wheat and summer-maize rotation cropland in the North China Plain. Agric. Ecosyst. Environ. 206, 33–45. doi: 10.1016/j.agee.2015.03.016 38826717

[B38] Wang K. Liu C. Zheng X. Pihlatie M. Li B. Haapanala S. . (2013). Comparison between eddy covariance and automatic chamber techniques for measuring net ecosystem exchange of carbon dioxide in cotton and wheat fields. Biogeosciences 10, 6865–6877. doi: 10.5194/bg-10-6865-2013

[B39] Wang Y. L. Wu P. N. Mei F. J. Ling Y. Qiao Y. B. Liu C. S. . (2021b). Does continuous straw returning keep China farmland soil organic carbon continued increase? A meta-analysis. J. Environ. Manage. 288, 112391. doi: 10.1016/j.jenvman.2021.112391 33823456

[B40] Whitley R. J. Macinnis-Ng C. M. O. Hutley L. B. Beringer J. Zeppel M. Williams M. . (2011). Is productivity of mesic savannas light limited or water limited? Results of a simulation study. Global Change Biol. 17, 3130–3149. doi: 10.1111/j.1365-2486.2011.02425.x 40046247

[B41] Whitley R. Medlyn B. Zeppel M. Macinnis-Ng C. Eamus D. (2009). Comparing the Penman–Monteith equation and a modified Jarvis–Stewart model with an artificial neural network to estimate stand-scale transpiration and canopy conductance. J. Hydrol. 373, 256–266. doi: 10.1016/j.jhydrol.2009.04.036 38826717

[B42] Wu F. Q. Tang Q. X. Cui J. P. Tian L. W. Guo R. S. Wang L. . (2024). Deficit irrigation combined with a high planting density optimizes root and soil water–nitrogen distribution to enhance cotton productivity in arid regions. Field Crops Res. 317, 109524. doi: 10.1016/j.fcr.2024.109524 38826717

[B43] Wutzler T. Lucas-Moffat A. Migliavacca M. Knauer J. Sickel K. Šigut L. . (2018). Basic and extensible post-processing of eddy covariance flux data with REddyProc. Biogeosciences 15, 5015–5030. doi: 10.5194/bg-15-5015-2018

[B44] Xiao C. Zhang F. C. Li Y. Fan J. L. Ji Q. Y. Jiang F. C. . (2024). Optimizing drip irrigation and nitrogen fertilization regimes to reduce greenhouse gas emissions, increase net ecosystem carbon budget and reduce carbon footprint in saline cotton fields. Agric. Ecosyst. Environ. 366, 108912. doi: 10.1016/j.agee.2024.108912 38826717

[B45] Yin Y. J. Ji D. C. Wang Y. Liu W. Y. Wang X. Liu K. S. . (2025). Effects of long-term cotton straw return on soil carbon and bacterial community in topsoil and deep soil. Agronomy 15, 1940. doi: 10.3390/agronomy15081940 30654563

[B46] Yu G. R. Wen X. F. Sun X. M. Tanner B. D. Lee X. Chen J. Y. (2006). Overview of ChinaFLUX and evaluation of its eddy covariance measurement. Agric. For. Meteorol. 137, 125–137. doi: 10.1016/j.agrformet.2006.02.011

[B47] Yue Z. W. Li Z. Yu G. R. Chen Z. Shi P. L. Qiao Y. F. . (2023). Seasonal variations and driving mechanisms of CO_2_ fluxes over a winter-wheat and summer-maize rotation cropland in the North China Plain. Agric. For. Meteorol. 342, 109699. doi: 10.1016/j.agrformet.2023.109699 38826717

[B48] Zhang W. Q. Dong A. H. Liu F. L. Niu W. Q. Siddique K. H. M. (2022). Effect of film mulching on crop yield and water use efficiency in drip irrigation systems: A meta-analysis. Soil Tillage Res. 221, 105392. doi: 10.1016/j.still.2022.105392 38826717

[B49] Zhang H. L. Luo H. H. Li L. Zhang Y. L. Zhang W. F. (2015). Characteristics of root and shoot biomass accumulation in high-yield cotton fields with mulch-drip irrigation. Cotton Sci. 27, 427–434. (in Chinese with English abstract).

[B50] Zhang Y. C. Shen Y. J. Xu X. L. Sun H. Y. Li F. Wang Q. (2013). Characteristics of the water–energy–carbon fluxes of irrigated pear (Pyrus bretschneideri Rehd) orchards in the North China Plain. Agric. Water Manage. 128, 140–148. doi: 10.1016/j.agwat.2013.07.007 38826717

[B51] Zhang Y. L. Tang Y. Q. Hu Y. J. Feng S. Y. Wang F. X. Wang Z. H. (2023). Effects of film mulching and soil wetted percentage of drip irrigation on soil hydrothermal conditions and sweet potato growth. Eur. J. Agron. 151, 126979. doi: 10.1016/j.eja.2023.126979 38826717

[B52] Zhou S. Q. Wang J. Liu J. X. Yang J. H. Xu Y. Li J. H. (2012). Evapotranspiration of a drip-irrigated, film-mulched cotton field in northern Xinjiang, China. Hydrol. Process. 26, 1169–1178. doi: 10.1002/hyp.8200 41531421

[B53] Zong R. Wang Z. H. Wu Q. Guo L. Lin H. (2020). Characteristics of carbon emissions in cotton fields under mulched drip irrigation. Agric. Water Manage. 231, 105992. doi: 10.1016/j.agwat.2019.105992 38826717

