# Supplementary material for: Biotic factors dominate carbon fluxes and budget of a drip-irrigated and plastic-mulched cotton system in an arid oasis of Northwest China
Source: Front Plant Sci. 2026 Aug 13;17:1884064. doi: 10.3389/fpls.2026.1884064 (PMC13519880; doi:10.3389/fpls.2026.1884064)
Supplement: Supplementary file 1 [file Table1.docx]

**Supplementary Materials**

**Table S1.** List of DIPM cotton system management practices

| Year | Cotton varieties | Fertilization  (N kg/ha) | Irrigation amount (mm) | Density (plant/m^2^) | Seed-cotton yield (kg/ha) |
| --- | --- | --- | --- | --- | --- |
| 2009 | Xinlu Zao-36 | 131.10 | 312.50 | 26.57 | 4500.00 |
| 2010 | Xinlu Zao-36 | 276.00 | 409.50 | 28.94 | 3900.00 |
| 2011 | Xinlu Zao-36 | 448.50 | 462.00 | 21.33 | 5145.00 |
| 2012 | Xinlu Zao-46 | 103.50 | 777.00 | 22.64 | 4200.00 |
| 2013 | Xinlu Zao-46 | 70.80 | 525.00 | 19.74 | 4200.00 |
| 2016 | 8026 | 310.50 | 420.00 | 26.08 | 4950.00 |
| 2017 | 8026 | 255.62 | 282.20 | 24.42 | 4800.00 |
| 2018 | Xinlu Zao-72 | 186.30 | 360.00 | 24.68 | 5250.00 |
| 2019 | Xinlu Zao-78 | 48.30 | 330.00 | 23.80 | 5475.00 |
| 2022 | Chuangmian 508 | 271.50 | 375.00 | 22.70 | 6150.00 |

Note: The pure nitrogen equivalent of nitrogen fertilizer is calculated as 0.46 × urea application amount + 0.18 × diammonium phosphate (DAP) + 0.12 × monoammonium phosphate (MAP).

**Table S2.** The information on five phases: emergence stage (ES), seedling stage (SS), squaring stage (BS), flowering stage (FS), boll opening stage (BOS) in the cotton seasons.

| Year | Emergence stage (ES) | | Seedling stage (SS) | | Squaring stage (BS) | Flowering stage (FS) | Boll opening stage (BOS) | Duration of the growing period (day) |
| --- | --- | --- | --- | --- | --- | --- | --- | --- |
|  | Sowing | Emergence | | Squaring | Flowering | Boll opening | Harves |  |
| 2009 | 04.12 | 04.26 | | 06.02 | 07.08 | 09.16 | 10.16 | 188 |
| 2010 | 04.28 | 05.08 | | 06.12 | 07.12 | 09.24 | 10.04 | 160 |
| 2011 | 04.19 | 05.04 | | 06.08 | 07.12 | 09.12 | 09.30 | 165 |
| 2012 | 04.17 | 04.28 | | 06.02 | 06.28 | 08.30 | 09.23 | 160 |
| 2013 | 04.08 | 04.26 | | 05.26 | 07.07 | 09.14 | 09.23 | 169 |
| 2016 | 04.08 | 04.20 | | 06.04 | 07.08 | 09.14 | 10.08 | 184 |
| 2017 | 04.12 | 04.26 | | 05.31 | 07.02 | 09.06 | 09.26 | 168 |
| 2018 | 04.16 | 04.28 | | 06.08 | 07.06 | 09.10 | 10.08 | 176 |
| 2019 | 04.15 | 04.30 | | 06.12 | 07.10 | 09.20 | 10.08 | 177 |
| 2022 | 04.16 | 04.28 | | 05.26 | 06.24 | 08.24 | 10.02 | 170 |

**Table S3.** Annual carbon fluxes (NEE, GPP, and R_eco_) of the DIPM cotton system for each study year (g C m⁻² yr⁻¹)

| Year | NEE | GPP | R_eco_ |
| --- | --- | --- | --- |
| 2009 | -285.81 | 882.41 | 596.49 |
| 2010 | -279.61 | 1022.94 | 743.40 |
| 2011 | -386.74 | 936.20 | 549.73 |
| 2012 | -388.15 | 1008.17 | 620.05 |
| 2013 | -311.75 | 1039.22 | 727.55 |
| 2016 | -375.80 | 943.16 | 567.90 |
| 2017 | -390.10 | 1041.62 | 651.60 |
| 2018 | -403.11 | 796.57 | 393.09 |
| 2019 | -313.53 | 1084.44 | 771.56 |
| 2022 | -424.85 | 902.07 | 477.63 |
| Mean ± SD | -355.95±50.02 | 965.68±84.45 | 609.90±113.89 |

**Appendix S1.** Detailed uncertainty analysis for NECB

This appendix provides detailed calculation procedures for the uncertainty analysis of the net ecosystem carbon budget (NECB) summarized in Section 4.4 of the main text. Three main sources of uncertainty were considered: (1) uncertainty in NEE gap‑filling propagated to NEP, (2) uncertainty in the root‑to‑shoot ratio (*R_r_s_*) used for NPP estimation, and (3) uncertainty in the straw return fraction (*f_R_*) used for C_straw_ calculation. Each source was quantified separately and then combined using first‑order error propagation to provide an overall uncertainty estimate for NECB.

**S1.1 Uncertainty from root‑to‑shoot ratio (*R_r_s_*) propagated to NECB**

The baseline *R_r_s_* was 0.11, based on destructive root sampling conducted at the WLWS_AES site in 2012 and 2013. NPP was calculated from aboveground biomass using equation (7) in the main text. C_straw_ was calculated using equation (6) in the main text. To quantify the sensitivity of NECB to uncertainty in *R_r_s_*, it was varied at ±10%, ±20%, and ±30% of its baseline value. These ranges were chosen based on observed variability across different yield levels and moisture conditions in Xinjiang cotton fields (Zhang et al., 2015; Gao et al., 2020). Notably, both studies were conducted at sites in the Shihezi area, which is located within the same oasis region as our WLWS_AES site and shares similar climatic conditions, soil types, and cotton management practices, making their reported *R_r_s_* variability directly relevant to our system.

When *R_r_s_* changes from its baseline value (0.11), the new NPP is:

${NPP}_{new}={NPP}_{baseline}\times\frac{1+R_{r\_s\_new}}{1+0.11}$	(S1)

The resulting change in C_straw_ and NECB is:

$\Delta NECB=\Delta C_{straw}=({NPP}_{new}-{NPP}_{baseline})\times f_{R}$	(S2)

Where NPP_baseline_ is the multi-year mean NPP (872.57 g C m⁻²), *R_r_s_* is the varied root-to-shoot ration (0.077-0.143 for ±30%), and *f_R_* is the baseline straw return fraction (0.85).

**S1.2 Uncertainty from straw return fraction (*f_R_*) propagated to NECB**

The baseline straw return fraction was 0.85, based on farmer surveys and agronomic records at the study site. To quantify the sensitivity of NECB to uncertainty in this parameter, *f_R_* was varied at 80%, 85%, and 90%, reflecting the typical interannual range reported for cotton straw incorporation in Xinjiang.

When *f_R_* changes from its baseline value, the change in C_straw_ and NECB is:

$\Delta NECB=\Delta C_{straw}=(NPP-C_{harvest})\times(f_{R_{new}}-0.85)$	(S3)

where NPP is the multi‑year mean NPP (872.57 g C m⁻²), C_harvest_ is the multi‑year mean carbon removed through seed‑cotton harvest (214.89 g C m⁻²), and f_R_*__new_* is the varied straw return fraction (0.80 or 0.90).

**S1.3 Combined uncertainty for NECB**

To provide an overall uncertainty estimate for NECB, the three main sources identified above were combined using first-order error propagation. For a function $Z=f(X_{1},X_{2}\ldots, X_{n})$ with independent input estimates *X_i_* having standard uncertainties *u*(*X_i_*), the combined standard uncertainty $u_{c}\left( Z \right)$:

$u_{c}\left( Z \right)=\sqrt{\sum_{i=1}^{n} \left( \frac{\partial f}{\partial X_{i}} \right)^{2}u^{2}(X_{i})}$	(S4)

Assuming independence among the three sources, the combined standard deviation for NECB is:

$\mathrm{SD}\left( \mathrm{NECB} \right)=\sqrt{{SD(NEP)}^{2}+{SD}_{f_{r\_s}}^{2}+{SD}_{f_{R}}^{2}}$	(S5)

where SD (NEP) is the standard deviation of NEP from NEE gap‑filling (50.02 g C m⁻², Table 4 in the main text), ${SD}_{f_{r\_s}}$ is the standard deviation of NECB from *R_r_s_* uncertainty (15.0 g C m⁻², from the ±30% scenario, Table S1.1), and ${SD}_{f_{R}}$ is the standard deviation of NECB from *f_R_* uncertainty (11.2 g C m⁻², from the 80%-90% scenarios, Table S1.1). The resulting uncertainty estimates are presented in Table 6 of the main text.

**Table S1.1.** Sensitivity of multi‑year mean NECB to uncertainties in root‑to‑shoot ratio (*R_r_s_*) and straw return fraction (*f_R_*).

| Parameter | Scenario | Parameter value | Change in NECB (%) | Change in NECB (g C m⁻²) |
| --- | --- | --- | --- | --- |
| Root-to-shoot ratio (*R_f_s_*) | Baseline | 0.11 | — | — |
|  | ±10% | 0.099–0.121 | ±1.5 | ±5.0 |
|  | ±20% | 0.088–0.132 | ±3.0 | ±10.0 |
|  | ±30% | 0.077–0.143 | ±4.5 | ±15.0 |
| Straw return fraction (*f_R_*) | Baseline | 0.85 | — | — |
|  | −5% | 0.80 | −3.4 | −11.2 |
|  | +5% | 0.90 | +3.4 | +11.2 |
| Most conservative combination | *R_r_s_* increased by 30% and *f_R_* = 80% | — | −7.3 | −24.3 |

Notes: Baseline multi‑year mean NECB = 332.64 g C m⁻². The most conservative combination yielded NECB = 308.40 g C m⁻². *R_f_s_* ranges were based on observed variability across different yield levels (Zhang et al., 2015) and moisture conditions (Gao et al., 2020). *f_R_* ranges were based on farmer surveys and agronomic records at the study site.

References for Appendix S1

Gao, H. Y., Li, J. H., Wang, Y. Y., et al. (2020). Response of photosynthetic characteristics and dry matter accumulation to drought of two cotton varieties with different drought resistance. Xinjiang Agric. Sci. 57, 233-244. doi: 10.6048/j.issn.1001-4330.2020.02.004 (in Chinese with English abstract).

Zhang, H. L., Luo, H. H., Li, L., et al. (2015). Characteristics of root and shoot biomass accumulation in high-yield cotton fields with mulch-drip irrigation. Cotton Sci. 27, 427-434. doi: 10.11963/issn.1002-7807.201505006 (in Chinese with English abstract).
